# Synergism of imipenem with fosfomycin associated with the active cell wall recycling and heteroresistance in *Acinetobacter calcoaceticus*-*baumannii* complex

**DOI:** 10.1038/s41598-021-04303-7

**Published:** 2022-01-07

**Authors:** Uthaibhorn Singkham-in, Tanittha Chatsuwan

**Affiliations:** 1 Department of Microbiology, Faculty of Medicine, Chulalongkorn University, Rama VI Road, Bangkok, 10330 Thailand; 2 Antimicrobial Resistance and Stewardship Research Unit, Faculty of Medicine, Chulalongkorn University, Rama VI Road, Bangkok, 10330 Thailand

**Keywords:** Microbiology, Molecular biology

## Abstract

The carbapenem-resistant *Acinetobacter calcoaceticus*-*baumannii* (ACB) complex has become an urgent threat worldwide. Here, we determined antibiotic combinations and the feasible synergistic mechanisms against three couples of ACB (*A. baumannii* (AB250 and A10), *A. pittii* (AP1 and AP23), and *A. nosocomialis* (AN4 and AN12)). Imipenem with fosfomycin, the most effective in the time-killing assay, exhibited synergism to all strains except AB250. MurA, a fosfomycin target encoding the first enzyme in the de novo cell wall synthesis, was observed with the wild-type form in all isolates. Fosfomycin did not upregulate *murA*, indicating the MurA-independent pathway (cell wall recycling) presenting in all strains. Fosfomycin more upregulated the recycling route in synergistic strain (A10) than non-synergistic strain (AB250). Imipenem in the combination dramatically downregulated the recycling route in A10 but not in AB250, demonstrating the additional effect of imipenem on the recycling route, possibly resulting in synergism by the agitation of cell wall metabolism. Moreover, heteroresistance to imipenem was observed in only AB250. Our results indicate that unexpected activity of imipenem on the active cell wall recycling concurrently with the presence of heteroresistance subpopulation to imipenem may lead to the synergism of imipenem and fosfomycin against the ACB isolates.

## Introduction

*Acinetobacter calcoaceticus*-*baumannii* (ACB) complex (particularly *A. baumannii*, *A. pittii*, and *A. nosocomialis*) has globally emerged as one of the most important nosocomial pathogens in healthcare settings^1^. A critical obstacle to the treatment of ACB infections is antibiotic resistance, especially carbapenem resistance^2^. Carbapenems are β-lactam antibiotics, which inhibit bacterial cell wall synthesis via covalent binding to penicillin-binding proteins (PBPs). The PBPs are enzymes (such as transpeptidase and transglycosylase) catalyzing peptidoglycan crosslinking^3^. The most dominant mechanism of carbapenem resistance in ACB is carbapenemase production, including Imipenemase (IMP), New Delhi Metallo-β-lactamases (NDM), Oxacillinase (OXA)-23, OXA-58, and OXA-24^4^. The secondary ones are the overexpression of efflux pumps (such as AdeB, AdeE, and AdeY) and the reduction of porins (including CarO, 33–36 kDa OMP, and OprD)^5,6^. Although colistin is effective against carbapenem-resistant ACB, unfortunately, colistin monotherapy is limited due to its toxicity. Therefore, antibiotic combinations, which are colistin-based or imipenem-based combinations, are inevitably used to combat carbapenem-resistant ACB^7,8^.

Fosfomycin inhibits the first step of the de novo cell wall (peptidoglycan) biosynthesis that converts uridine diphosphate *N*-acetylglucosamine (UDP-GlcNAc) to enolpyruvyl-UDP-GlcNAc by UDP-*N*-acetylglucosamine enolpyruvyl transferase (MurA). Subsequently, enolpyruvyl-UDP-GlcNAc catalyzed by MurA is converted to UDP *N*-acetylmuramic acid (UDP-MurNAc) by UDP-*N*-acetylenolpyruvoylglucosamine reductase (MurB). The UDP-MurNAc is a precursor in cell wall synthesis. Fosfomycin covalently binds to MurA^9^, resulting in the inactivation of cell wall synthesis^9^. Fosfomycin is recommended not only for uncomplicated urinary tract infections (UTIs) by *Escherichia coli* but also for the complicated UTIs caused by extended-spectrum beta-lactamase (ESBL)-producing Enterobacteriaceae^10^. The potent activity of fosfomycin has been announced in combination with antibiotics against non-fermentative bacteria, including *Pseudomonas aeruginosa* and *A. baumannii*^11,12^. Many mechanisms of fosfomycin resistance have been characterized, including mutation at the active site of MurA and overexpression of fosfomycin-specific efflux pump, named AbaF^9,13^. Moreover, *P. aeruginosa* and *A. baumannii* are intrinsically resistant to fosfomycin by cell wall recycling pathway^14,15^. This bypath is a MurA-independent pathway, leading to fosfomycin resistance. Besides the de novo pathway, UDP-MurNAc is also synthesized through cell wall recycling. Briefly, the recycling route begins with the inner membrane transporter, AmpG, uptakes shedding cell wall, anhydromuropeptides^14,15^. The anhydromuropeptides (GlcNAc-AnhMurNAc-Ala-Gln-DAP-Ala) are converted to GlcNAc and AnhMurNAc by β-*N*-acetylglucosaminidase (NagZ) and AnhMurNAc-l-alanine amidase (AmpD), respectively. The AnhMurNAc is phosphorylated by AnhMurNAc kinase (AnmK), generating MurNAc-6P. The MurNAc-6P is dephosphorylated to yield MurNAc by MurNAc-6P phosphatase (MupP). The C1 hydroxyl group of MurNAc is phosphorylated by MurNAc kinase (AmgK), yielding MurNAc-α-1P that is converted to cell wall precursor, UDP-MurNAc, by *N*-acetylmuramate α-1-P uridylyltransferase (MurU). We previously reported that imipenem in combination with fosfomycin was effective against carbapenem-resistant *A. baumannii*^16^. However, the synergism was not associated with carbapenem resistance mechanisms. Here, we investigated the in vitro activity of antibiotic combinations against carbapenem-resistant ACB complex, including *A. pittii* and *A. nosocomialis*^17^. To clarify the plausible synergistic mechanisms of imipenem and fosfomycin combination, the difference in the resistance mechanisms of both imipenem and fosfomycin were investigated among synergistic and non-synergistic strains. For carbapenem resistance, production of carbapenemases, overexpression of efflux pumps, and reduction of OMPs were characterized. For fosfomycin resistance, mutation and expression of MurA, overexpression of efflux pump, and expression of cell wall recycling enzymes were evaluated. In addition, the presence of heteroresistance to either imipenem or fosfomycin was performed among the ACB isolates. The heteroresistance is defined as a heterogeneous bacterial population, which has diverse antibiotic resistance potency linking to antibiotic treatment failure^16^. We hypothesized that the heteroresistance phenotype may be related to a failure of imipenem and fosfomycin combination (no synergism). In this study, the heteroresistance characteristics were identified by using a population analysis profile (PAP) assay.

## Results

### Antibiotic susceptibility and carbapenemase genes in ACB isolates

The two couples of *A. baumannii* (AB250 and A10) and *A. pittii* (AP1 and AP23), unique clones emerging in our hospital, and a pair of *A. nosocomialis* (AN4 and AN12) were included in this study. Among all six carbapenem-resistant ACB isolates, both *A. baumannii* AB250 and A10 carried *bla*_OXA-24_, both *A. nosocomialis* AN4 and AN12 carried *bla*_OXA-23_, and *A. pittii* AP1 and AP23 carried *bla*_OXA-58_ with *bla*_IMP_ and *bla*_OXA-23_, respectively (Table 1). The antibiotic susceptibilities were interpreted following the Clinical and Laboratory Standards Institute (CLSI) guidelines (Supplementary Table S1). All ACB isolates were susceptible to amikacin but no intermediate resistant or resistant to fosfomycin (Table 1). All isolates were intermediate to colistin with the MICs below the resistant breakpoint (4 mg/L) (Table 1).

**Table 1:** The minimum inhibitory concentrations (MICs) of six ACB isolates to imipenem (IPM), meropenem (MEM), fosfomycin (FOF), amikacin (AMK), and colistin (CT) were determined by agar dilution method.

ACB species	Isolate	MIC (mg/L)					Carbapenemase gene	Allelic number							Sequence type (ST)
		IPM	MEM	FOF	AMK	CT		*gltA*	*gyrB*	*gdhB*	*recA*	*cpn60*	*gpi*	*rpoD*	
*A. baumannii*	AB250	16 (R)	16 (R)	128 (I)	4 (S)	1 (I)	*bla*_OXA-51_, *bla*_OXA-24_	1	12	56	1	1	177	26	1416
*A. baumannii*	A10	128 (R)	256 (R)	256 (R)	2 (S)	2 (I)	*bla*_OXA-51_, *bla*_OXA-24_	1	15	13	12	4	163	5	1426
*A. pittii*	AP1	32 (R)	32 (R)	256 (R)	0.5 (S)	2 (I)	*bla*_OXA-58_, *bla*_IMP_	56	104	137	7	25	153	74	1419
*A. pittii*	AP23	16 (R)	32 (R)	128 (I)	2 (S)	1 (I)	*bla*_OXA-23_	56	104	137	7	51	153	74	1420
*A. nosocomialis*	AN4	16 (R)	32 (R)	256 (R)	2 (S)	2 (I)	*bla*_OXA-23_	39	65	142	30	25	114	28	958
*A. nosocomialis*	AN12	32 (R)	64 (R)	128 (I)	2 (S)	2 (I)	*bla*_OXA-23_	39	65	142	30	25	114	28	958

The results were interpreted as susceptible (S), intermediate resistant (I), or resistant (R). The presence of carbapenemase genes was performed by PCR. The clonality was performed by MLST Oxford scheme as seven allelic numbers and a sequence type number.

### Clonal of six ACB isolates

The clonality of all six ACB isolates was studied by the multi-locus sequence typing (MLST) Oxford scheme (Table 1). *A. baumannii* and *A. pittii* isolates belonged to different clones (ST types). Both *A. baumannii* AB250 and A10 carrying *bla*_OXA-24_ belonged to ST1416 and ST1426, respectively. *A. pittii* AP1 and AP23 carrying different carbapenemase genes belonged to different clones as ST1419 and ST1420, respectively, which differed only in an allelic number of *cpn60*. *A. nosocomialis* AN4 and AN12 belonged to the same ST, ST958.

### OMP profiles

**Figure 1:**
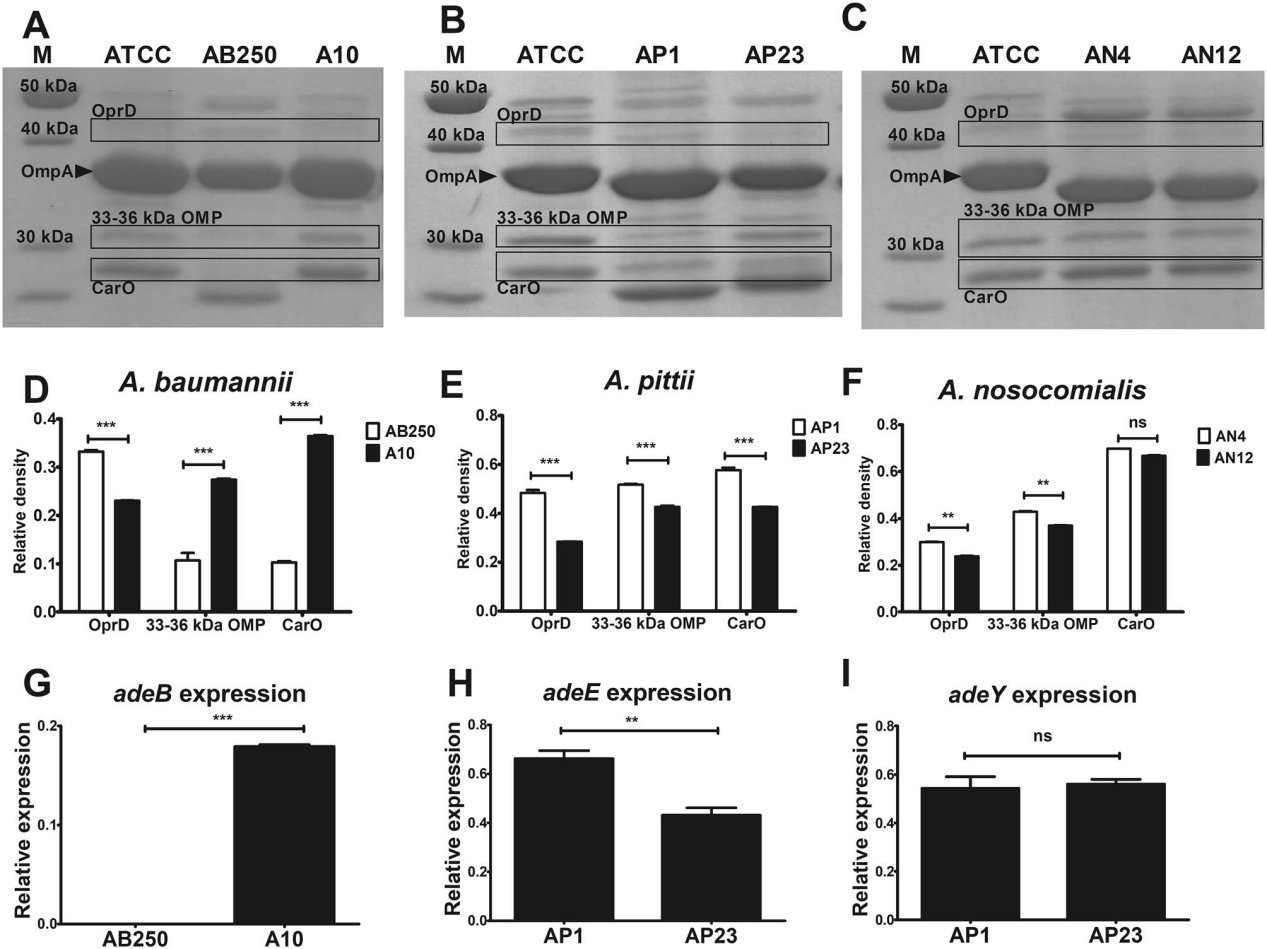
OMP profiles, OMP expression, and efflux pump gene expression among six ACB isolates. (**A**–**C**) OMP extracts were studied by SDS-PAGE. (**D**–**F**) The relative density of OMP expression was calculated and normalized to OmpA. All experiments were performed in triplicate. Mean values of the relative density were plotted with error bars representing the standard error of the mean (n = 3). The *p*-values were calculated using unpaired two-tailed t-test (*p-value ˂0.05; **p-value < 0.01; ***p-value < 0.001 and ns, non-significant). (**G**) The relative mRNA expression of *adeB* among *A. baumannii* was evaluated by RT-PCR and normalized to 16S rRNA expression. The relative mRNA expression of *adeE* (**H**) and *adeY* (**I**) was evaluated by RT-PCR and normalized to 16S rRNA expression. All experiments were performed in triplicate. Mean values of the relative mRNA expression were plotted with error bars representing the standard error of the mean (n = 3). The *p*-values were calculated using unpaired two-tailed t-test (*p-value ˂0.05; **p-value < 0.01; ***p-value < 0.001 and ns, non-significant).

Three OMPs act as porins for carbapenem entry, including CarO (29 kDa OMP), 33–36 kDa OMP, and OprD (43 kDa OMP). These OMPs were found in all OMP profiles of six ACB isolates (Fig. 1A–C, Supplementary Fig. S1A–C). The relative density of each OMP was calculated and normalized to that of major OMP (OmpA) as internal control, representing OMP expression (Fig. 1D–F). OMP expressions were compared within the species. The reduction of 33–36 kDa OMP and CarO expression was observed in *A. baumannii* AB250, but the reduction of OprD was observed in A10 (Fig. 1A,D). In *A. pittii*, the reduction of OprD, 33–36 kDa OMP, and CarO was observed in AP23 (Fig. 1B,E). Among *A. nosocomialis* isolates, the reductions of OprD and 33–36 kDa OMP were observed in AN12 (Fig. 1C,F).

### Overexpression of efflux pumps

Multidrug efflux pumps play one of the essential roles in the antibiotic resistance of ACB isolates. The overexpression of efflux pump phenotype for carbapenems was characterized by using CCCP. No ACB isolate showed the positive phenotype of efflux pump overexpression to carbapenems (Table 2). Therefore, we determined the expression level of efflux pump genes. In this study, *A. baumannii* carried *adeB* gene, whereas *A. pittii* carried *adeE* and *adeY* genes (Table 2). Neither of these genes was found in *A. nosocomialis* isolates (Table 2). Overexpression of *adeB* was observed in *A. baumannii* A10, which had a high level of carbapenem MICs (128–256 mg/L) (Fig. 1G and Table 1). *A. pittii* AP1 showed slightly overexpressed *adeE* (Fig. 1H) with twofold carbapenem MICs above these of AP23 (Table 1). *A. pittii* AP1 and AP23 equally displayed the *adeY* expression (Fig. 1I).

**Table 2:** MICs of imipenem (IPM), meropenem (MEM), and fosfomycin (FOF) in either with or without CCCP and the presence of efflux pump gene of six ACB isolates.

ACB species	Isolate	MIC (mg/L)						Efflux pump gene			
		IPM	IPM + CCCP	MEM	MEM + CCCP	FOF	FOF + CCCP	*adeB*	*adeE*	*adeY*	*abaF*
*A. baumannii*	AB250	16	16	16	16	128	64	+	−	−	+
*A. baumannii*	A10	128	128	256	256	256	128	+	−	−	+
*A. pittii*	AP1	32	32	32	32	256	256	−	+	+	ND
*A. pittii*	AP23	16	16	32	32	128	128	−	+	+	ND
*A. nosocomialis*	AN4	16	16	32	32	256	128	−	−	−	ND
*A. nosocomialis*	AN12	32	32	64	32	128	128	−	−	−	ND

The positive result for efflux overexpression was defined as the decreased MICs at least fourfold at the presence of CCCP. The presence of efflux pump genes was determined by PCR. (+) presence of gene, (−) absence of gene, ND not determined.

In conclusion of carbapenem resistance mechanisms, the major mechanism found in all isolates was carbapenemase production. OXA-23 production was present in AP23, AN4, and AN12. OXA-24 production was present in AB250 and A10. The production of OXA-58 with IMP was found in AP1. For the reduction of porins, reduced OprD was present in A10, AP23, and AN12. Reduced 33–36 kDa porin was present in AB250, AP23 and AN12. Reduced CarO was present in AB250 and AP23. For efflux pumps, overexpression of *adeB* and *adeE* was present in A10 and AP1, respectively.

### Activity of antibiotic combinations against six ACB isolates

The in vitro activities of imipenem or meropenem in combination with either amikacin, colistin, or fosfomycin against *A. baumannii*, *A. pittii*, and *A. nosocomialis* isolates were determined by checkerboard assay. The MICs of antibiotic combinations that used for the fractional inhibitory concentration (FIC) index calculation in Eq. (1), are present in Supplementary Table S2. The most effective combination was imipenem with fosfomycin that exhibited synergism (FICI ≤ 0.5) against all six ACB isolates (Table 3). Secondly, meropenem plus fosfomycin and imipenem plus amikacin were potential combinations against *A. nosocomialis* and *A. pittii* (Table 3). No synergism was observed in imipenem plus colistin, meropenem plus amikacin, and meropenem plus colistin. From the results of fosfomycin susceptibility, fosfomycin alone had inadequate potency against all ACB isolates (Table 1). The combination results differed in that fosfomycin had a synergistic activity with carbapenems, especially imipenem.

**Table 3:** The activity of carbapenems, imipenem (IPM) and meropenem (MEM), in combination with amikacin (AMK), colistin (CT), or fosfomycin (FOF) performed by checkerboard assay.

ACB species	Isolate	FIC index (interpretation)					
		IPM + AMK	IPM + CT	IPM + FOF	MEM + AMK	MEM + CT	MEM + FOF
*A. baumannii*	AB250	0.75 (N)	2.00 (N)	0.5 (S)	1.00 (N)	0.63 (N)	1.00 (N)
*A. baumannii*	A10	0.75 (N)	0.75 (N)	0.5 (S)	1.00 (N)	1.00 (N)	0.75 (N)
*A. pittii*	AP1	0.5 (S)	1.00 (N)	0.38 (S)	1.00 (N)	0.56 (N)	0.5 (S)
*A. pittii*	AP23	0.75 (N)	1.00 (N)	0.5 (S)	0.63 (N)	1.00 (N)	1.00 (N)
*A. nosocomialis*	AN4	0.63 (N)	2.00 (N)	0.5 (S)	0.75 (N)	0.75 (N)	0.5 (S)
*A. nosocomialis*	AN12	0.38 (S)	1.00 (N)	0.5 (S)	0.75 (N)	2.00 (N)	0.5 (S)

The FIC index was calculated and interpreted as synergism (S) and no interaction (N).

### Time-killing curves of imipenem with fosfomycin against six ACB isolates

**Figure 2:**
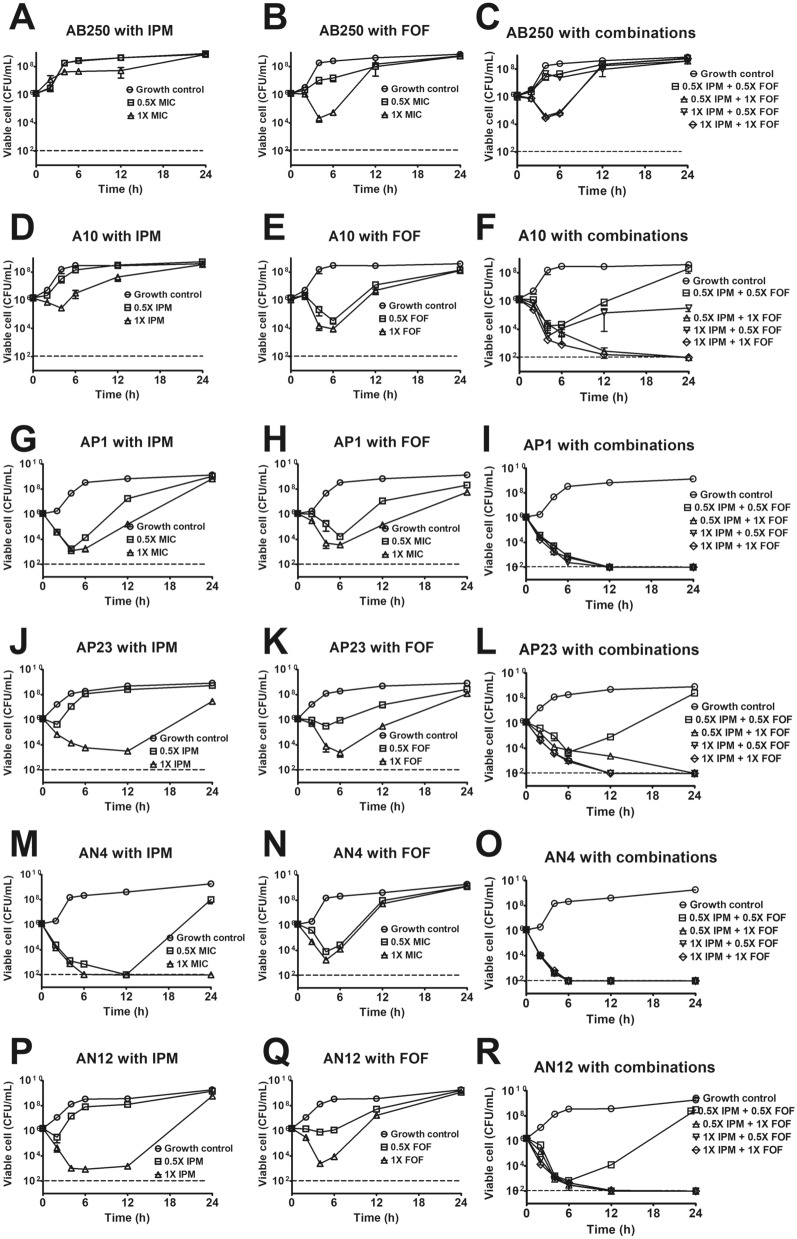
Time-killing curves of imipenem with fosfomycin against six ACB isolates. There were three conditions, including 0.5× MIC and 1× MIC of imipenem alone (**A**,**D**,**G**,**J**,**M**,**P**), 0.5× MIC and 1× MIC of fosfomycin alone (**B**,**E**,**H**,**K**,**N**,**Q**), and the combinations (**C**,**F**,**I**,**L**,**O**,**R**) Mean values of viable cells were plotted with error bars representing the standard error of the mean (n = 3). All experiments were performed in triplicate and the detection limit of the viable cells is 10^2^ CFU/mL (dash lines).

As a result of the checkerboard assay, we, therefore, verified the synergism of imipenem with fosfomycin against six ACB isolates by time-killing assay. In every ACB isolate, the growth control curves were normal S-curves, which reached the log phase (exponential phase) at 2 to 4 h of incubation (Fig. 2). Both 0.5× and 1× imipenem MICs were unable to kill *A. baumannii* AB250 (Fig. 2A) and A10 (Fig. 2D). In the presence of 0.5× fosfomycin MIC, AB250 was able to grow (Fig. 2B), but it was killed by 1× fosfomycin MIC for 4 h and regrew subsequently (Fig. 2B). Both fosfomycin concentrations (0.5× and 1× MICs) killed A10 for 6 h before the regrowth (Fig. 2E). No combination was able to achieve the synergistic activity with AB250 (Fig. 2C). AB250 was not killed by either 0.5× or 1× imipenem MIC combined with 0.5× fosfomycin MIC (Fig. 2C). Although imipenem in combination with 1× fosfomycin MIC eliminated AB250, the regrowth occurred after 4 h, resulting in no synergism. In contrast, most combinations were able to eradicate A10 leading to synergism, except 0.5× combination that achieved regrowth and no synergism (Fig. 2F).

In *A. pittii*, both concentrations of single imipenem killed AP1 for 4 h before regrowth occurring (Fig. 2G). Although 0.5× imipenem MIC did not kill AP23, 1× MIC was able to eradicate for 12 h before regrowth (Fig. 2J). All fosfomycin concentrations alone could kill AP1 for 6 h, then regrowth occurred (Fig. 2H). Whereas 0.5× and 1× fosfomycin MICs showed inhibitory activity and bactericidal activity, respectively, to AP23 for 6 h ahead of regrowth (Fig. 2K). All combinations eradicated AP1 that reached an undetectable limit, resulting in synergism (Fig. 2I). In AP23, no synergism was observed in 0.5× imipenem and 0.5× fosfomycin MIC combination that regrowth appeared after 6 h (Fig. 2L). Apart from this combination, others killed AP23, achieving undetectable points and synergistic activity (Fig. 2L).

In *A. nosocomialis*, both concentrations of imipenem alone killed AN4 for 12 h ahead of regrowth occurring in the 0.5× imipenem MIC (Fig. 2M). In contrast to AN12, 0.5× imipenem MIC could not kill, whereas the regrowth after 12 h was observed in the 1× imipenem MIC (Fig. 2P). All fosfomycin concentrations alone were able to kill AN4 before regrowth appeared at 4 h (Fig. 2N). Killing and inhibition of AN12 were observed by 1× and 0.5× fosfomycin MIC, respectively, before regrowth (Fig. 2Q). AN4 and AN12 were killed by all combinations, resulting in synergistic activity (Fig. 2O,R), except 0.5× imipenem with 0.5× fosfomycin MIC that regrowth appeared in AN12 after 6 h (Fig. 2R).

### MurA amino acid sequences among six ACB isolates

To understand fosfomycin resistance mechanisms in ACB isolates, the amino acid sequences of the fosfomycin target, MurA, were determined and analyzed. Amino acid sequences of the MurA among six ACB isolates are shown in Supplementary Fig. S2. All MurA sequences were wild-type (WT) that displayed no mutation associated with fosfomycin resistance, including Cys116, Lys22, Arg121, Arg398, Asp370, and Leu371 (arrows in Supplementary Fig. S2).

### Expression of *murA* gene in six ACB isolates

**Figure 3:**
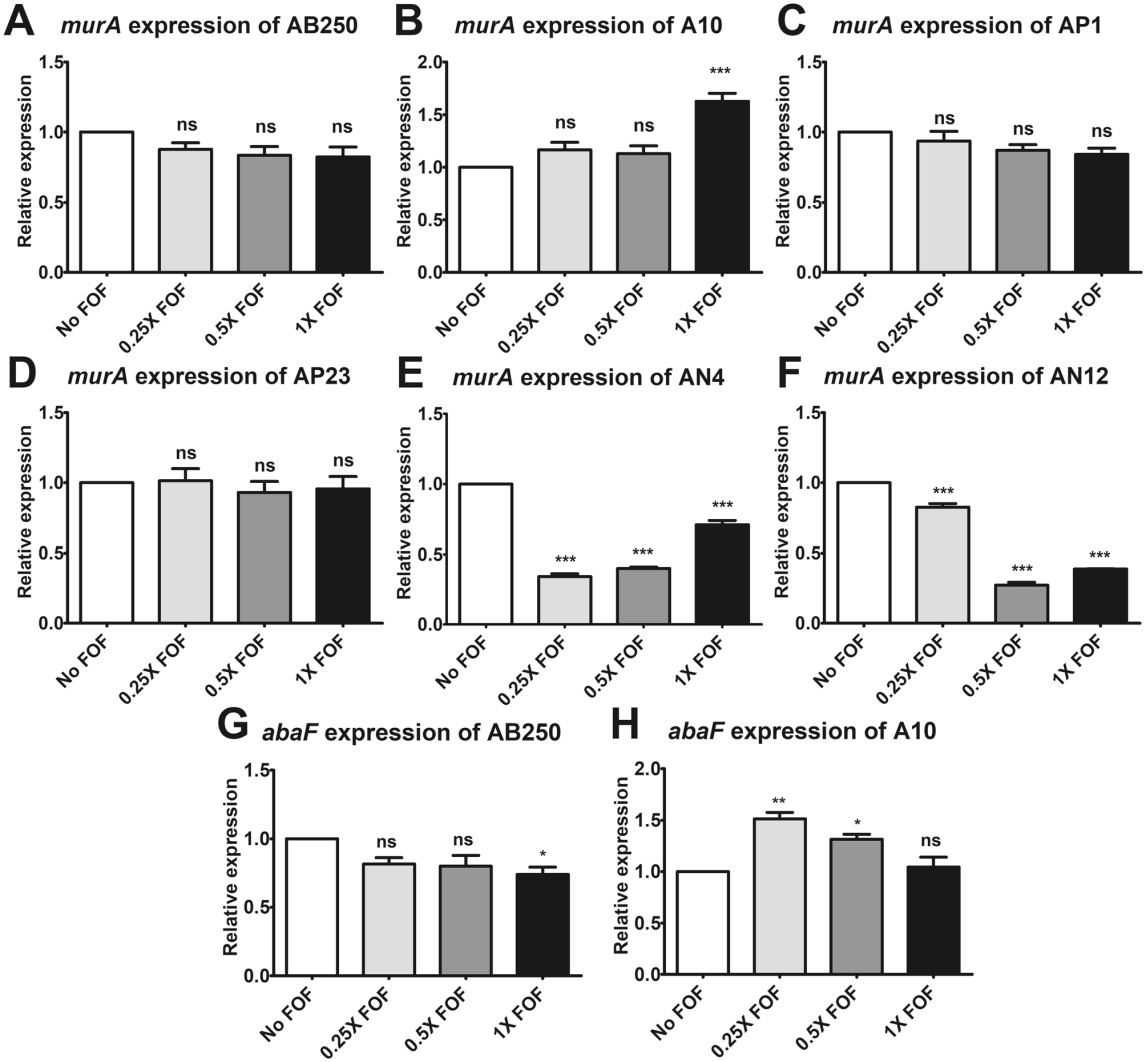
Relative mRNA expression of *murA* in six ACB isolates. RT-PCR assay of *murA* expression after 2 h of exposure to fosfomycin was determined in *A. baumannii* AB250 (**A**) and A10 (**B**), *A. pittii* AP1 (**C**) and AP23 (**D**), and *A. nosocomialis* AN4 (**E**) and AN12 (**F**). The relative mRNA expressions at each condition (in the presence of 0.25× MIC, 0.5× MIC, or 1× MIC of fosfomycin) were normalized to 16S rRNA expression and compared to the mRNA expression level of each isolate in the absence of fosfomycin. The relative mRNA expressions of *abaF* in *A. baumannii* AB250 (**G**) and A10 (**H**) at each condition (in the presence of 0.25× MIC, 0.5× MIC, or 1× MIC of fosfomycin) were normalized to 16S rRNA expression and compared to the mRNA expression level of each isolate in the absence of fosfomycin. All experiments were performed in triplicate. Mean values of the relative mRNA expression were plotted with error bars representing the standard error of the mean (n = 3). The *p*-values were calculated using one-way ANOVA, Dunnett’s multiple comparison test (*p-value ˂0.05; **p-value < 0.01; ***p-value < 0.001 and ns, non-significant).

Interestingly, no MurA mutation was found in all ACB isolates that intermediate or resistant to fosfomycin. To determine whether MurA was associated with fosfomycin resistance in ACB isolates, *murA* expression was evaluated in the presence of fosfomycin for 2 h. Fosfomycin did not affect *murA* expression in most isolates, including *A. baumannii* AB250 (Fig. 3A), A10 (Fig. 3B), *A. pitti* AP1 (Fig. 3C), and AP23 (Fig. 3D). Additionally, fosfomycin (0.5× MIC) had no impact on *murA* expression in these isolates at 6 and 12 h after exposure (Supplementary Fig. S3A–D). However, 1× fosfomycin MIC upregulated *murA* expression in A10 (Fig. 3B). In contrast, fosfomycin significantly downregulated *murA* expression in *A. nosocomialis* AN2 (Fig. 3E) and AN14 (Fig. 3F). In addition, 0.5× MIC fosfomycin downregulated *murA* expression after exposure for 6 and 12 h in *A. nosocomialis* (Supplementary Fig. S3E,F). These results indicate that fosfomycin does not affect the de novo cell wall synthesis via MurA in *A. baumannii* and *A. pittii* isolates but in *A. nosocomialis* isolates.

### Overexpression of efflux pump induced by fosfomycin

Another mechanism of fosfomycin resistance reported in *A. baumannii* is the overexpression of the efflux pump, AbaF. Firstly, the phenotype of efflux pump overexpression was performed using CCCP. Unfortunately, all isolates showed negative phenotypes of efflux pump overexpression (Table 2). Therefore, the level of *abaF* expression was determined by RT-PCR. Fosfomycin exhibited the downregulation of *abaF* in *A. baumannii* AB250 (Fig. 3G). Overexpression of *abaF* was observed when using a low concentration of fosfomycin against *A. baumannii* A10 (Fig. 3H). These results indicate that overexpression of *abaF* may involve fosfomycin susceptibility in a strain-specific manner in *A. baumannii*.

### Cell wall recycling pathway in six ACB isolates

Our results suggest other mechanisms that bypass the MurA-dependent cell wall synthesis pathway. We screened several enzyme genes that play a role in the cell wall recycling pathway. All *A. baumannii* and *A. nosocomialis* carried all tested genes, including *ampG*, *nagZ*, *anmK*, *amgK*, and *murU*, whereas *amgK* did not detect in both *A. pittii* isolates (Table 4).

**Table 4:** The presence of cell wall recycling enzyme genes in six ACB isolates. (+) presence of gene, (**−**) absence of gene.

ACB species	Isolate	Cell wall recycling gene					
		*murA*	*ampG*	*nagZ*	*anmK*	*amgK*	*murU*
*A. baumannii*	AB250	+	+	+	+	+	+
*A. baumannii*	A10	+	+	+	+	+	+
*A. pittii*	AP1	+	**+ **	+	+	−	+
*A. pittii*	AP23	+	+	+	+	−	+
*A. nosocomialis*	AN4	+	+	+	+	+	+
*A. nosocomialis*	AN12	+	+	+	+	+	+

**Figure 4:**
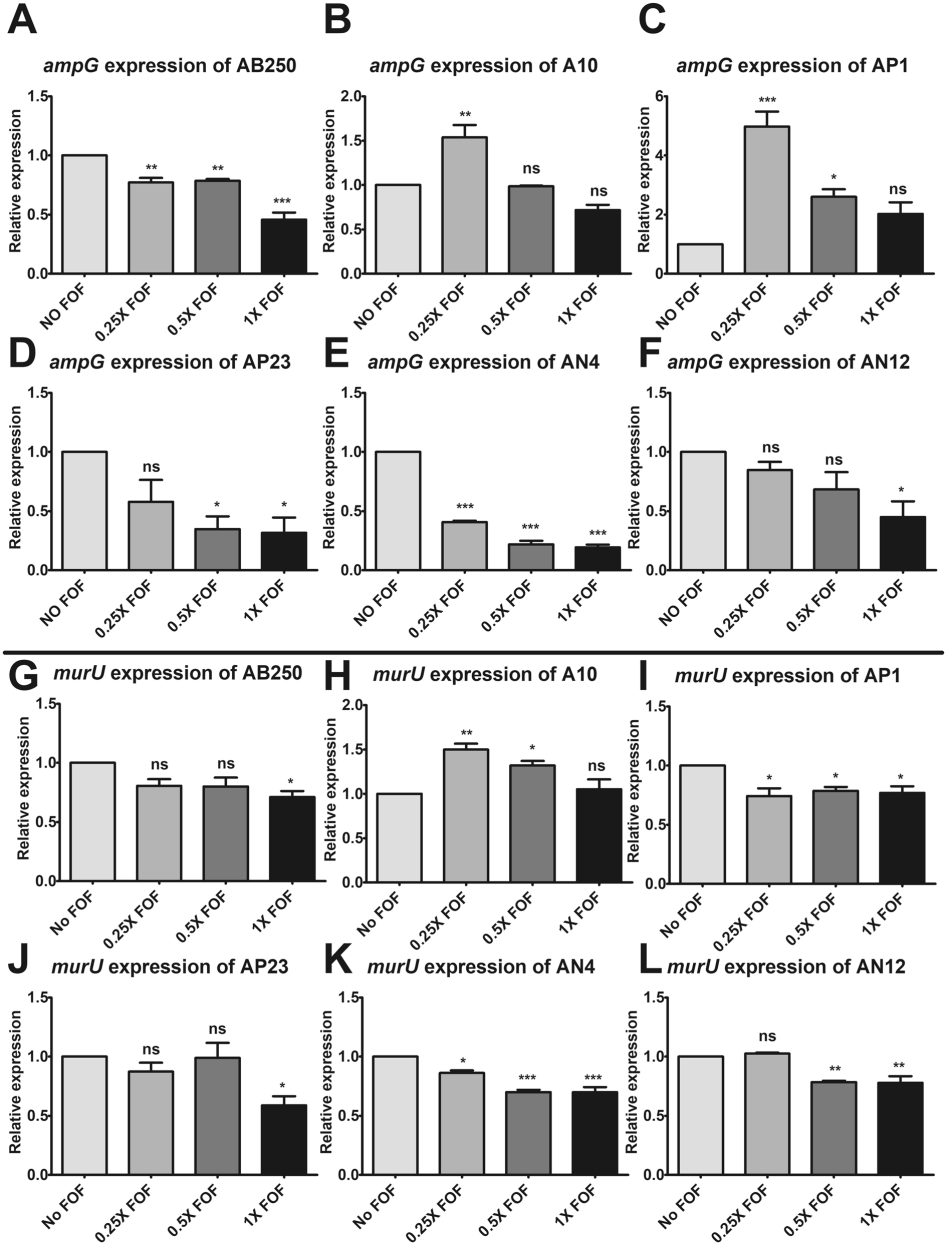
Relative mRNA expression of *ampG* in the presence of fosfomycin was determined by RT-PCR assay in *A. baumannii* AB250 (**A**) and A10 (**B**), *A. pittii* AP1 (**C**) and AP23 (**D**), and *A. nosocomialis* AN4 (**E**) and AN12 (**F**). Relative mRNA expression of *murU* in the presence of fosfomycin was determined by RT-PCR assay in *A. baumannii* AB250 (**G**) and A10 (**H**), *A. pittii* AP1 (**I**) and AP23 (**J**), and *A. nosocomialis* AN4 (**K**) and AN12 (**L**). The relative mRNA expressions at each condition (in the presence of 0.25× MIC, 0.5× MIC, or 1× MIC of fosfomycin) were normalized to 16S rRNA expression and compared to the mRNA expression level of each isolate in the absence of fosfomycin. All experiments were performed in triplicate. Mean values of the relative mRNA expression were plotted with error bars representing the standard error of the mean (n = 3). The *p*-values were calculated using one-way ANOVA, Dunnett’s multiple comparison test (*p-value ˂0.05; **p-value < 0.01; ***p-value < 0.001 and ns, non-significant).

The initial step of cell wall recycling is the uptake of shedding peptidoglycan into the cytoplasm through the AmpG transporter. Therefore, the expression level of *ampG* was determined in the presence of fosfomycin for 2 h. Fosfomycin dose-dependently downregulated *ampG* expression in *A. baumannii* AB250 (Fig. 4A), AP23 (Fig. 4D), *A. nosocomialis* AN4 (Fig. 4E), and AN12 (Fig. 4F). In contrast, fosfomycin less than the MICs significantly upregulated *ampG* expression in *A. baumannii* A10 (Fig. 4B) and *A. pittii* AP1 (Fig. 4C).

**Figure 5:**
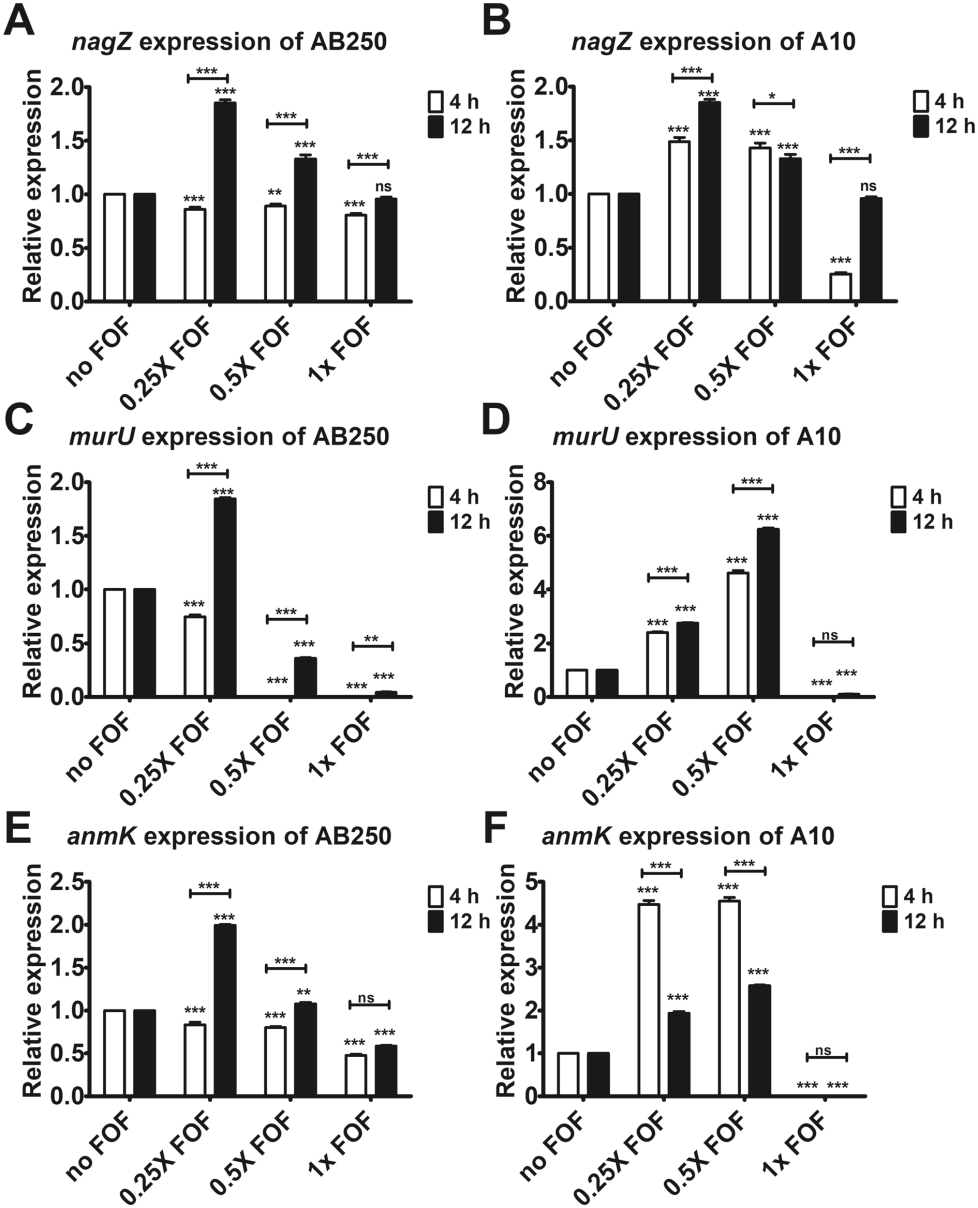
Relative mRNA expression of *nagZ*, *murU*, and *anmK* in the presence of fosfomycin for 4 and 12 h determined by RT-PCR assay in *A. baumannii* AB250 (**A**,**C**,**E**) and A10 (**B**,**D**,**F**). The relative mRNA expressions at each condition (in the presence of 0.25× MIC, 0.5× MIC, or 1× MIC of fosfomycin) were normalized to 16S rRNA expression and compared to the mRNA expression level of each isolate in the absence of fosfomycin. All experiments were performed in triplicate. Mean values of the relative mRNA expression were plotted with error bars representing the standard error of the mean (n = 3). The *p*-values were calculated using one-way ANOVA, Dunnett’s multiple comparison test (*p-value ˂0.05; **p-value < 0.01; ***p-value < 0.001 and ns, non-significant).

Another essential protein in cell wall recycling is MurU, the last enzyme producing the cell wall precursor that bypasses the MurA-dependent pathway. Downregulation of *murU* by fosfomycin was found in most ACB isolates (Fig. 4G,I–L) except *A. baumannii* A10, which overexpressed *murU* by a low level of fosfomycin (Fig. 4H). In summary, according to *ampG* and *murU* expression, fosfomycin did not upregulate cell wall recycling at 2 h of exposure in ACB isolates (Fig. 4). This is in accordance with the time-kill results, which demonstrated that no increase of bacterial cells in 2 h of fosfomycin exposure (Fig. 2). Except for AP1, fosfomycin induced *ampG* expression (Fig. 4C), but downregulated *murU* expression (Fig. 4I). Subsequently, we focused on *A. baumannii* AB250 and A10, which were non-synergistic and synergistic strains, respectively, by the combination of fosfomycin and imipenem. To evaluate the difference between these isolates, the expression of the additional genes in the cell wall recycling, including *nagZ*, *murU*, and *anmK*, was conducted at 4 and 12 h of exposure to fosfomycin, the rebound in growth (regrowth) occurred (Fig. 2B,E). The different expression patterns were significantly observed at 4 h (Fig. 5). The downward trend of expression was found in AB250, in which fosfomycin slightly downregulated *nagZ* (Fig. 5A) and *anmK* (Fig. 5E) but remarkably reduced *murU* expression (Fig. 5C). Unlikely, the upward trend of expression was present in A10, in which fosfomycin underneath the MICs significantly increased *nagZ* (Fig. 5B), *murU* (Fig. 5D), and *anmK* (Fig. 5F) expression. These results suggest that the active cell wall recycling of A10 is superior to that of AB250 in the presence of fosfomycin.

Both isolates exhibited disparate expression between 4 and 12 h. AB250 showed significant upregulation of *nagZ* (Fig. 5A), *murU* (Fig. 5C), and *anmK* (Fig. 5E) in the presence of fosfomycin beneath the MICs at 12 h. This pattern was similar to that of A10, which increased *nagZ* (Fig. 5B), *murU* (Fig. 5D), and *anmK* (Fig. 5F) expression. These results indicate the elevation of cell wall recycling activity at 12 h in both isolates. Notably, *murU* (Fig. 5C,D) and *anmK* (Fig. 5E,F) had either little or no expression in the presence of fosfomycin MIC, although viable cells of both isolates were above 10^6^ CFU/mL at 12 h (Fig. 2B,E).

**Figure 6:**
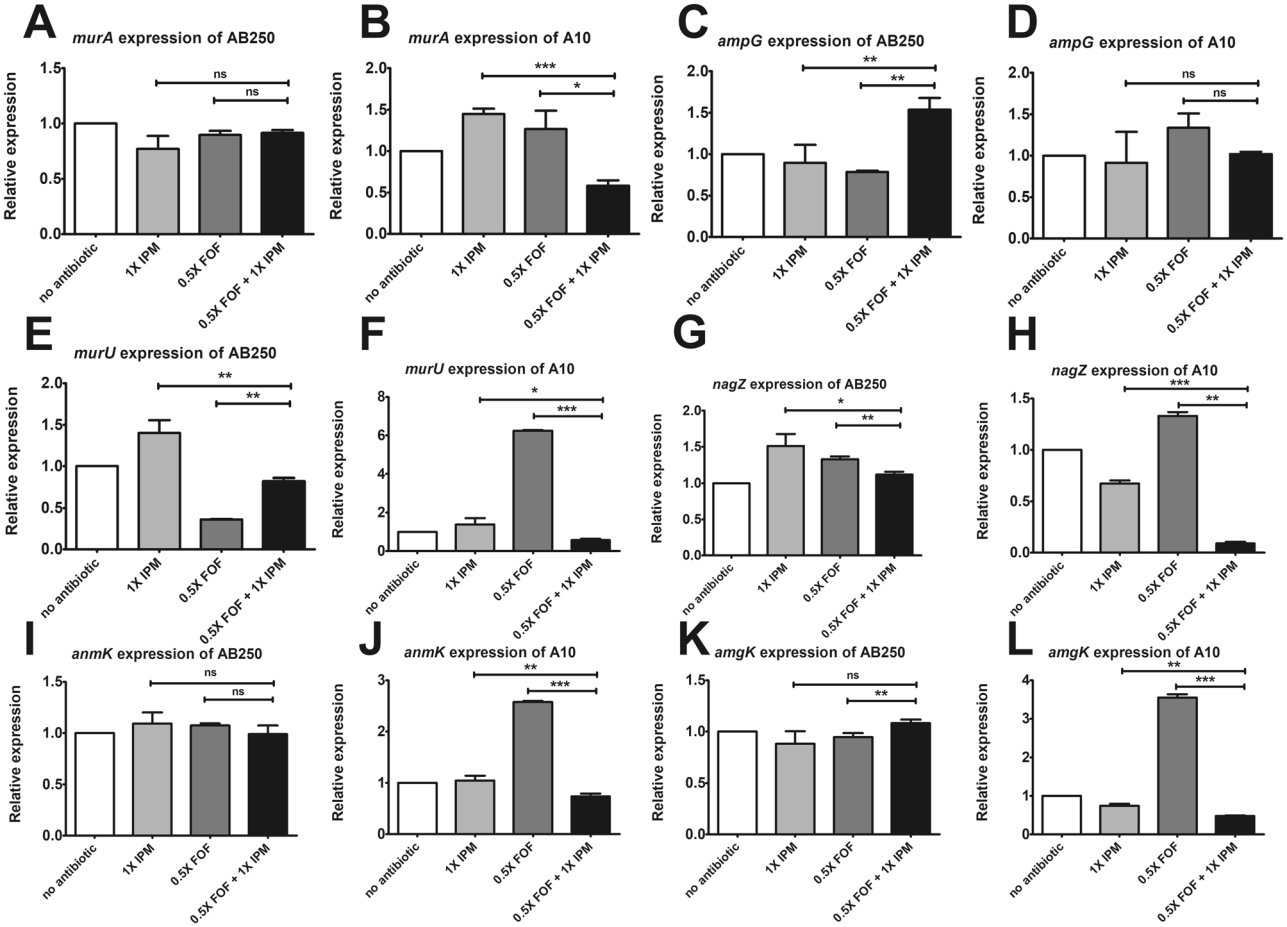
Relative mRNA expression of *murA* (**A**,**B**), *ampG* (**C**,**D**), *murU* (**E**,**F**), *nagZ* (**G**,**H**), *anmK* (**I**,**J**), and *amgK* (**K**,**L**) in the absence of antibiotic, the presence of 0.5× fosfomycin MIC, and the combination of 0.5× fosfomycin MIC with 1× imipenem MIC for 12 h determined by RT-PCR assay. The relative mRNA expressions at each condition were normalized to 16S rRNA expression and compared to the mRNA expression level of each isolate in the absence of antibiotic. All experiments were performed in triplicate. Mean values of the relative mRNA expression were plotted with error bars representing the standard error of the mean (n = 3). The *p*-values were calculated using one-way ANOVA, Dunnett’s multiple comparison test (*p-value ˂0.05; **p-value < 0.01; ***p-value < 0.001 and ns, non-significant).

To evaluate the role of imipenem combination on cell wall recycling, the expression of cell wall recycling was determined after 12 h exposure to 0.5× fosfomycin MIC with 1× imipenem MIC, which had synergistic activity in A10 (Fig. 2F) but not in AB250 (Fig. 2C), compare to either fosfomycin or imipenem alone. Imipenem in the combination did not affect *murA* expression in AB250 (Fig. 6A) but downregulated in A10 (Fig. 6B). The transporter gene, *ampG*, was upregulated in the imipenem combination in AB250 (Fig. 6C) but had no effect in A10 (Fig. 6D). In AB250, although imipenem combination induced *murU* expression, its expression level was slightly lower than that in the absence of imipenem (Fig. 6E). In contrast, the combination reduced *murU* expression in A10 nearly to that in the absence of any antibiotic (Fig. 6F). Interestingly, imipenem combination significantly downregulated *nagZ* (Fig. 6H), *anmK* (Fig. 6J), and *amgK* (Fig. 6L) in A10 compared to control and either single fosfomycin or imipenem. However, the combination showed a few effects on these gene expressions in A250 (Fig. 6G,I,K). These results indicate that imipenem may affect at least in part of cell wall recycling resulting in synergism with fosfomycin.

**Figure 7:**
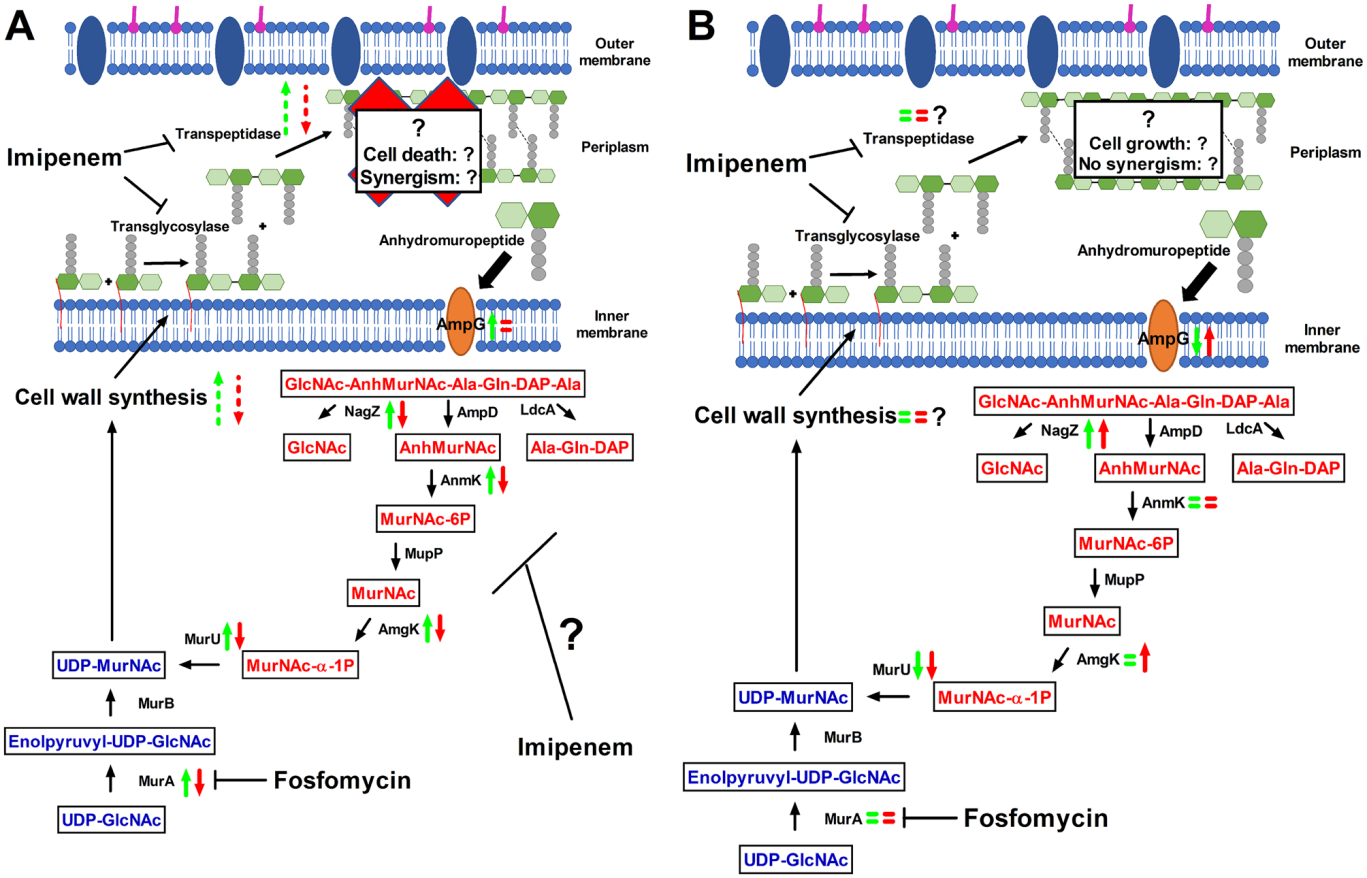
Summary of cell wall recycling gene expression and proposed mechanism of synergistic mechanism of imipenem and fosfomycin combination in *A. baumannii* A10 (**A**) and AB250 (**B**). The cell wall recycling expressions in Fig. 6 were summarized in this scheme. Green and red symbols are represented the gene expression in the presence of 0.5× fosfomycin MIC and in the combination of 0.5× fosfomycin MIC and 1× imipenem MIC, respectively. Upward arrows, downward arrows, and equal arrows are represented upregulation, downregulation, and not change of expression compared to without antibiotic, respectively. Question marks and dash arrows are represented the proposed results that did not determined. Red words in the boxes are represented metabolites in the cell wall recycling pathway. Blue words in the boxes are represented metabolites in the de novo cell wall synthesis.

The summary of cell wall recycling and the proposed synergistic mechanism of fosfomycin and imipenem in AB250 and A10 are present in Fig. 7. Imipenem synergistically reduced the expression of cell wall recycling in A10 (red symbols in Fig. 7A), indicating dwindling cell wall synthesis that may result in cell death. Differently, imipenem showed a minor effect on the alteration of cell wall recycling (red symbols in Fig. 7B), indicating a sluggish but functional and adequate cell wall synthesis that may result in cell growth.

### PAP assay

**Figure 8:**
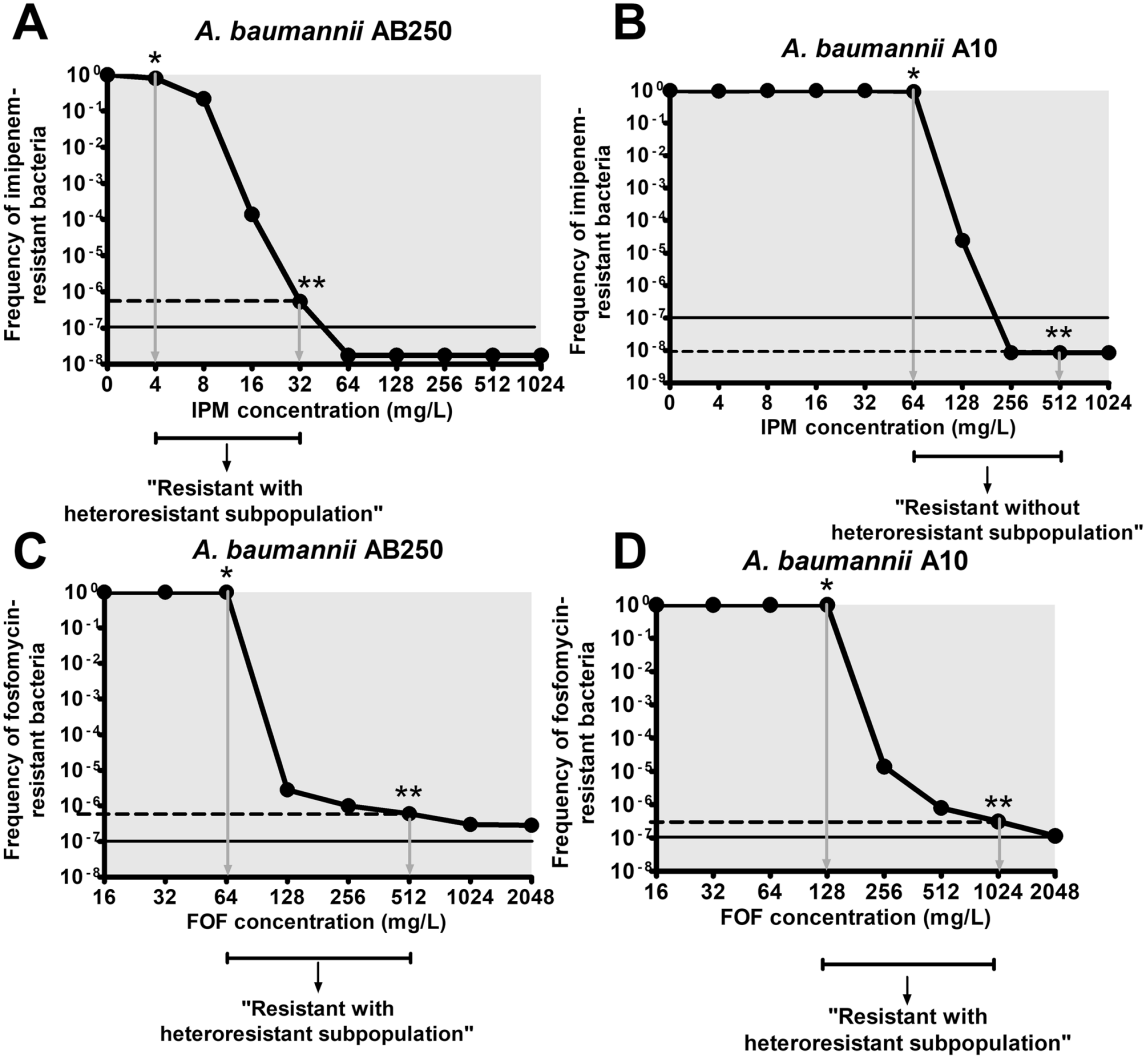
PAP of imipenem and fosfomycin in *A. baumannii* AB250 (**A**,**C**) and A10 (**B**,**D**). The frequency of antibiotic-resistant bacteria was the relative of viable cells at each antibiotic concentration normalized to those at the absence of antibiotic. All experiments were performed in triplicate. Mean values of the frequency of antibiotic-resistant bacteria were plotted with error bars representing the standard error of the mean (n = 3). The “resistant with heteroresistant subpopulation” was defined as that the frequency of antibiotic-resistant bacteria at eightfold above the resistance level of the main population (dash lines) was higher than 10^–7^. *: antibiotic concentration (the resistance level) of the main population, **: eightfold above antibiotic concentration (the resistance level) of the main population.

In addition to antibiotic resistance mechanisms, the population phenotypes of all ACB isolates were evaluated. The phenotypes of populations were determined by using the PAP study, which displayed the frequency of bacteria growing on agar supplemented with various concentrations of tested antibiotics, which calculated by using Eq. (2). The positive of heteroresistant subpopulation was defined as the presence of bacterial frequency that grows above 10^–7^ on the agar supplemented with eightfold above the antibiotic concentration of the main population. For imipenem, AB250 exhibited the heteroresistant subpopulation in which the frequency of bacteria was above 10^–7^ at eightfold above the antibiotic concentration of the main population (32 mg/L) (Fig. 8A). This phenotype was called resistant with heteroresistant subpopulation to imipenem (Fig. 8A). In the case of A10, the frequency of the growth at eightfold above the resistance level of the main population to imipenem (512 mg/L) was less than 10^–7^, indicating no heteroresistant subpopulation (Fig. 8B). Therefore, A10 was resistant without heteroresistant subpopulation to imipenem (Fig. 8B). No heteroresistant subpopulation to imipenem was also observed in *A. pittii* AP1 (Supplementary Fig. S4A), AP23 (Supplementary Fig. S4B), *A. nosocomialis* AN4 (Supplementary Fig. S4C), and AN12 (Supplementary Fig. S4D).

For fosfomycin, all isolates exhibited the resistant frequency upper the cut-off point (10^–7^) at eightfold above the antibiotic concentration of the main population of each isolate (Fig. 8C,D, Supplementary Fig. S4E–H). Therefore, all ACB isolates were resistant with heteroresistant subpopulations to fosfomycin. In summary, among all isolates, *A. baumannii* AB250 was the only isolate that imipenem and fosfomycin combination had no synergistic activity and was resistant with heteroresistant subpopulation to imipenem. These results may indicate the feasible association between synergism and the heteroresistant subpopulation. However, further study is required to understand the impact of heteroresistance in antibiotic synergism.

## Discussion

ACB complex, generally considered saprophytes, has been regarded as a critical multidrug-resistant nosocomial pathogen in clinical settings within the last two decades^18^. Although *A. pittii* and *A. nosocomialis* are lower grade pathogens than *A. baumannii*, their abilities to resistant to antibiotics, particularly carbapenems, have been reported^17,19,20^. Moreover, the emergence of colistin-resistant ACB is rising worldwide^21^; fortunately, no colistin-resistant isolate was observed in our study. A couple of *A. baumannii* and *A. pittii* in our study belonged to new STs submitted to the PubMLST^22^, indicating unique clones emerging in our hospital. Carbapenem-resistant *A. nosocomialis* belonged to ST958 which is similar to carbapenem-resistant *A. baumannii* isolated from a patient in Uruguay, indicating a close relation among the ACB complex^23^. The vast majority of ACB complex produce class D carbapenemases, including OXA-23, OXA-24, OXA-58, and OXA-51. The latter is an intrinsic carbapenemase in *A. baumannii*; however, OXA-51-type carbapenemase is transferred to non-*baumannii Acinetobacter* via horizontal transfers particularly, plasmid^24^. Reduction of 33–36 kDa OMP and CarO was found in *A. pittii* AP23 and *A. nosocomialis* AN4, respectively, whereas the loss of OprD is reported in *A. nosocomialis* isolated from Taiwan, and no reduction of OMP is found in non-*baumannii Acinetobacter* isolated from South Korea^25,26^. Interestingly, not only involved in antibiotic resistance, but these porins also act as virulence factors^27^. Moreover, we found overexpression of *adeB* and *adeE* in *A. baumannii* and *A. pittii*, respectively. A report from Taiwan revealed that carbapenem-resistant *A. nosocomialis* exhibited overexpression of *adeB*, leading to tigecycline resistance^28^. *A. baumannii* may act as the coach supporting antibiotic resistance determinants and virulence machines to other species in the ACB complex. Not surprisingly, non-*baumannii Acinetobacter* is turning into a potential pathogen.

Due to the limitation of antibiotic usage for carbapenem-resistant ACB, many studies focused on antibiotic combinations, most of which were colistin-based combinations such as imipenem or meropenem plus colistin and sulbactam plus colistin^29–31^. Interestingly, sulbactam is a beta-lactamase inhibitor, not an antibiotic, showed synergism with colistin against carbapenem-resistant ACB^29^. A report revealed that sulbactam inhibits penicillin-binding proteins (PBPs), including PBP1 and PBP3^32^. Another non-traditional antibiotic being effective in the combinations is fosfomycin. Fosfomycin displayed synergism with sulbactam and colistin against carbapenem-resistant *A. baumannii*^33,34^. Moreover, the synergy of fosfomycin in the combination with imipenem was revealed in our previous study^16^. Therefore, we determined the in vitro activity of fosfomycin plus imipenem and other combinations and characterized the resistance mechanisms to unveil the plausible synergistic mechanism. Apart from *A. baumannii*, the synergistic activity was also found against *A. pittii* and *A. nosocomialis*. This study demonstrates that imipenem with fosfomycin could be used for combating carbapenem-resistant ACB complex. However, the effectiveness of the combination should be clinically evaluated further.

Fosfomycin inhibits peptidoglycan synthesis by covalently binding to MurA (Fig. 7). Generally, fosfomycin is recommended for UTIs caused by certain Enterobacteriaceae, notably *E. coli*^10^. Nevertheless, *E. coli* is resistant to fosfomycin by various mechanisms. Firstly, the alteration of drug target, MurA, generally occurs at the active site, Cys115, and the ligand-binding site, including Lys22, Arg120, and Arg397 (Supplementary Fig. S2)^35^. This mechanism has never been found in *A. baumannii*, so MurA has been inspired to be a new target^36^. Unfortunately*, A. baumannii* normally has a high level of fosfomycin susceptibility with WT MurA, suggesting the intrinsic resistance by the MurA-independent pathway^37^. The second mechanism is the mutations of fosfomycin transporters (GlpT and UhpT), leading to decreased uptake of fosfomycin, but this mechanism has not been revealed in *A. baumannii*^37^. Thirdly, the production of fosfomycin-modifying enzymes (such as FosA, FosB, FosC, and FosX) is the most frequently found mechanism in both Gram-negative and Gram-positive bacteria^37^. *fosA* has been deposited on 2% and 7.8% of the *A. baumannii* and *A. pittii* genomes, respectively, demonstrating that FosA production is not an intrinsic resistance mechanism in ACB^38^. Another mechanism reported in *A. baumannii*, is the efflux transporter, AbaF^13^. The deletion of *abaF* confers the reduction of eightfold fosfomycin MIC in *A. baumannii*^13^. In our study, both *A. baumannii* had nearly equal fosfomycin MICs, but overexpression of *abaF* was found in one isolate, indicating a minor role of the AbaF. An additional mechanism being debate is the cell wall recycling pathway that bypasses the de novo synthesis via MurA (Fig. 7)^14^. The cell wall recycling pathway has been well characterized in *E. coli*, but it differs from that of other Gram-negative bacteria, including *A. baumannii* that is the MurA-bypass pathway^15,39,40^.

Although fosfomycin induces overexpression of *murA* in *E. coli*^41^, most ACB isolates, whose MurA exhibited wild-type enzymes that susceptible to fosfomycin, were not induced *murA* expression by fosfomycin except at a high concentration (1× MIC) in A10 (Fig. 3B). These results with the presence of genes encoding cell wall recycling enzymes strongly suggest the cell wall recycling pathway that is a MurA-independent pathway in the ACB complex. In spite the fact that AmpG transporter upregulates at a high level of substrates, indicating an active turnover of the cell wall^42^, most ACB isolates were no change or downregulation of *ampG* together with *murU* after 2 h treated by fosfomycin, except the low fosfomycin concentration in A10 (Fig. 4B) and AP1 (Fig. 4C). Downregulations of these genes indicate the downward trend of cell wall syntheses that are related to inhibition of cell growth in the time-kill curves (Fig. 2).

According to the time-kill curves with fosfomycin, the log phase of growth shifted to 4–12 h instead of 2–4 h. Therefore, the cell wall expression in *A. baumannii* AB250 (no synergistic strain) and A10 (synergistic strain) were focused at 4 and 12 h. Downward expression of the recycling was found in non-synergistic strain, whereas the upward trend was observed in synergistic strain, indicating the more active recycling in the synergistic strain. However, both strains exhibited upregulation of the recycling in 12 h compared to that of 4 h. Unexpectedly in both isolates, fosfomycin equal to the MICs barely induced *murU* expression (Fig. 5C,D), but the viability of growth did not affect (Fig. 5B,E), possibly due to achieving the steady-state of the recycling at that time. In addition to being the last enzyme in the cell wall recycling, MurU is believed that plays an important role in the preservation of a steady-state of a MurNAc pool and the suppression of an anhMurNAc pool^43^. According to these hypotheses, the bacterial growth with shutting down of *murU* at 12 h exposure to fosfomycin is probably caused by the excess of cell wall materials. It is the limitation of our study that the bacterial metabolites were not determined.

In combination of 0.5× fosfomycin with 1× imipenem that did not affect bacterial growth (Fig. 2A–F), imipenem showed a tiny role on the recycling expression in non-synergistic strain, indicating an inert response of AB250. In contrast, the significant downregulation of the recycling was observed in synergistic strain, suggesting enhance effect of imipenem in perturbation of the recycling of A10. Therefore, imipenem may be synergistic with fosfomycin at least in part downregulation of the cell wall recycling.

The targets of imipenem are PBPs, not the cell wall recycling, thus imipenem indirectly plays a role on the recycling. The cell wall synthesis is composed of the precursor production (the de novo or recycling bypath) and peptidoglycan crosslinking (by the PBPs). Both processes are inevitably related and sophisticated. Many studies are supporting the hypothesis that imipenem not only inhibits PBPs, but also interrupts other cellular metabolisms^44,45^. For instance, mecillinam, whose targets are PBPs, simultaneously blocks the PBPs and enhances a cycle of cell wall synthesis and turnover via the Rod system, resulting in depleting PG precursor pools in *E. coli*. Similar to a report in *A. baumannii*^46^, imipenem affects not only PBP2 and PBP1a, but also perturbs the Rod system. Therefore, imipenem in combination with fosfomycin may complicatedly disturb cell wall metabolism, at least in some parts, resulting in decreasing of cell wall recycling.

An additional factor that may additionally affect the activity of antibiotic combination, is heteroresistance. The heteroresistance is a phenotype of a subpopulation that displays a greater potency of antibiotic resistance than that in the main population. The higher-level resistance in the heteroresistance is caused by the mutation of antibiotic resistance determinants^47^. Therefore, the ACB isolates may differently express the cell wall recycling to generate the fosfomycin heteroresistance. Furthermore, the bacterial regrowth in the time-kill curves may be the growth of fosfomycin and imipenem heteroresistance subpopulations. However, regrowth may be due to loss of antibiotic stability. Imipenem has a half-life of 0.7 h in serum in vitro^48^, whereas fosfomycin has a half-life of 5.7 h in plasma^9^. There are various methods for the detection of the heteroresistant subpopulation. The gold standard method is the PAP assay^49^. In this study, the PAP assay was used with the addition of the frequency of heteroresistant subpopulations as recommended by Andersson et al.^47^. Among six ACB isolates, only *A. baumannii* AB250, non-synergistic strain, exhibited heteroresistant to imipenem (Fig. 8A), whereas all isolates had heteroresistant subpopulations to fosfomycin (Fig. 8C,D, Supplementary Fig. S3E–H). Notably, all isolates resistant with heteroresistant subpopulations to fosfomycin showed similar patterns of the time-kill curves that regrowth occurred after 4 h, possibly due to subpopulation change. However, all ACB isolates exhibited unstable heteroresistance phenotypes in which subcultures with antibiotic-free media (> 50 generations)^47,50^ showed the loss of heteroresistance (Supplementary Table S3). Therefore, the unstable heteroresistance may lead to failure treatment by combination therapy and is difficult to detect by the routine method.

In summary, although, most ACB isolates possessed cell wall recycling pathway, their response to fosfomycin were quite difference, indicating strain-specific responses. The synergistic strain (A10) exhibited more active of the cell wall recycling than no synergistic strain (AB250). Imipenem, in the combination, significantly downregulated the cell wall recycling in the synergistic strain, indicating the additional action apart from inhibition of PBPs. Therefore, the feasible synergistic mechanism of imipenem and fosfomycin was an unexpected function of imipenem that affects at least in part of cell wall recycling resulting in synergism via downregulation of cell wall recycling concurrently without heteroresistance subpopulation. Nevertheless, the role of heteroresistance in the synergism of imipenem and fosfomycin is still unclear and needs further investigation. Moreover, both cell wall metabolism and bacterial response to antibiotics are dynamic and sophisticated. Therefore, the comparative metabolic perturbations of these strains should be further investigated to unveil the synergistic mechanism of imipenem and fosfomycin. This study demonstrates the in vitro synergism of imipenem with fosfomycin against carbapenem-resistant ACB complex.

## Methods

### Bacterial strains and antibiotic susceptibility testing

Two *A. baumannii* isolates (AB250 and A10), two *A. pittii* isolates (AP1 and AP23), and two *A. nosocomialis* isolates (AN4 and AN12) from our previous study were clinical strains that were isolated from an individual patient at the King Chulalongkorn Memorial Hospital, Bangkok, Thailand^16,17^. All strains were routinely tested for antibiotic susceptibility according to CLSI recommendation^51^. Susceptibility of imipenem (Apollo Scientific), meropenem (Sigma-Aldrich), amikacin (Sigma-Aldrich), and colistin (Sigma-Aldrich) was performed by broth microdilution method using cation-adjust Mueller–Hinton broth (CAMHB) (Becton Dickenson BBL) whereas that of fosfomycin was performed by agar dilution method using Mueller–Hinton agar (MHA) (Becton Dickenson BBL) supplemented with 25 mg/L of glucose-6-phosphate (G6P) (Sigma-Aldrich). Bacterial reference strains were *E. coli* ATCC 25922 and *P. aeruginosa* ATCC 27853. The antibiotic susceptibility was interpreted according to the CLSI guideline^51^ (Supplementary Table S1).

Moreover, phenotype of overexpression of efflux pump against imipenem, meropenem, and fosfomycin was performed by agar dilution method compared with the addition of carbonyl cyanide m-chlorophenyl hydrazone (CCCP) (Sigma-Aldrich), a proton coupler interrupting efflux pump function. The positive phenotype of overexpression of efflux pump was defined as at least fourfold decreased of antibiotic MIC (minimum inhibitory concentration) observed in the presence of CCCP.

### MLST

The clonal relationship among all six ACB isolates was studied by MLST as recommendation of the PubMLST^22^. Briefly, the partial fragments of seven housekeeping genes, including *gltA*, *gyrB*, *gdhB*, *recA*, *cpn60*, *gpi*, and *rpoD*, were amplified from extracted genomic DNA by PCR using primer recommended by the PubMLST^22^. The PCR products were sequenced. We performed and analyzed the MLST profiles according to the MLST Oxford scheme. The allelic numbers of each gene and the sequence type (ST) numbers were obtained from the PubMLST.

### OMP study

OMPs of all isolates were separated by using ultracentrifugation method as our previous study^16^. Briefly, the mid-log phase bacterial cells were broken by sonication and the membrane fractions were collected by ultracentrifugation at 100,000*g* for 1 h at 4 °C (Beckman-Coulter). OMPs were extracted by using 2% sodium *N*-lauryl sarconate (Merck Millipore) and collected by ultracentrifugation again at 100,000*g* for 1 h at 4 °C. The OMPs were resuspended with phosphate buffer saline (Sigma Aldrich) and were quantified the concentration by using Bio-Rad protein assay (Bio-Rad). The OMP profiles were studied by SDS-PAGE. The concentration of OMPs loaded in each well was 10 µg. The gels were stained with coomassie brilliant blue, dried on cellophane sheets, and captured by using an image scanner. The density of each protein band was determined by using ImageJ. The relative density of each protein was calculated and compared to that of control protein, OmpA, in the same bacterial isolate.

### Detection of antibiotic resistance genes, efflux pump genes, and cell wall recycling genes

Antibiotic resistance genes (including *bla*_OXA-51_, *bla*_OXA-24_, *bla*_OXA-23_, and *bla*_IMP_), efflux pump genes (including *adeB*, *adeE*, *adeY*, and *abaF*) and cell wall recycling genes (including *murA*, *ampG*, *nagZ*, *anmK*, *amgK*, and *murU*) were detected by PCR. The primers used in this study are shown in Supplementary Table S4.

### Expression level of efflux pump genes and cell wall recycling genes

The expression level of efflux pump genes (including *adeB*, *adeE*, *adeY* and *abaF*) and cell wall recycling genes (including *murA*, *ampG*, *nagZ*, *anmK*, *amgK*, and *murU*) was performed by RT-PCR. The total RNA of the ACB isolates was extracted by using Trizol reagent (Invitrogen) and converted to cDNA by using RevertAid First Strand cDNA Synthesis Kit (Thermo Scientific). The relative expression of each gene was normalized with 16S rRNA expression. This experiment was performed in triplicate. The primers used in this study are shown in Supplementary Table S4.

### Checkerboard assay

In vitro activity of carbapenem (imipenem and meropenem) in combination with either amikacin, fosfomycin, or colistin against all six ACB isolates was performed by checkerboard assay as our previous study^16^. Briefly, the checkerboard assay was conducted in 96-well microtiter plates in which the rows contained CAMHB supplemented with serial dilution of one antibiotic and the columns contained CAMHB supplemented with serial dilution of another antibiotic. The plates were inoculated with the ACB isolates and incubated at 37 °C for 18–24 h. The MICs of each antibiotic in alone and the combination were read by naked eyes. The FIC (fractional inhibitory concentration) index was calculated using the following equation:

1 $$FICI=\frac{MIC \; of \; drug \; A \; in \; combination}{MIC \; of \; drug \; A \; alone}+\frac{MIC \; of \; drug \; B \; in \; combination}{MIC \; of \; drug \; B \; alone}$$

The results were interpreted as following: synergism as FICI ≤ 0.5, no interaction as 0.5 < FICI ≤ 4, and antagonism as FICI > 4.

### Time-killing assay

Synergism of the most effective combination, imipenem with fosfomycin, was confirmed by time-killing assay as our previous study^16^. The concentrations of 1× MIC and 0.5× MIC of imipenem and fosfomycin were tested as a single agent and in combination. The flask contained CAMHB supplemented with each concentration of single imipenem or fosfomycin or the combinations was inoculated with 10^6^ CFU/mL of ACB isolates. Nine conditions of antibiotic were tested, including growth control (no antibiotic), 0.5× imipenem MIC, 1× imipenem MIC, 0.5× fosfomycin MIC, 1× fosfomycin MIC, 0.5× imipenem + 0.5× fosfomycin MIC, 0.5× imipenem MIC + 1× fosfomycin MIC, 1× imipenem MIC + 0.5× fosfomycin MIC, and 1× imipenem MIC + 1× fosfomycin MIC. The flasks contained fosfomycin were supplemented with 25 mg/L of G6P. All flasks were incubated at 37 °C for 24 h with shaking at 120 rpm. The viable cells at 0, 2, 4, 6, 12, and 24 h after incubation were counted and plotted. The synergism was defined as at least 2log decreased of viable cells compared to the most active single agent after 24 h of incubation. The bactericidal activity was defined as at least 3log decreased of viable cells compared to the viable cells of initial inoculation. This experiment was performed in triplicate.

### Population analysis profile (PAP) assay

The characteristics of ACB population that composed of either homogeneous or heterogeneous populations were determined by PAP assay^47^. Briefly, overnight culture of bacterial strains was serially diluted and plated on MHA agar containing different antibiotic concentrations (at 0, 4, 8, 16, 32, 64, 128, 256, 512, and 1024 mg/L of imipenem and at 0, 16, 32, 64, 128, 256, 512, 1024, and 2048 mg/L of fosfomycin supplemented with 25 mg/L of G6P). After 24 h of incubation, the viable cells on each plate were counted. The frequency of resistant cells was calculated using the following equation:

2 $$Frequency \; of \; resistant \; cells=\frac{Viable \; cells \; on \;MHA \;with \;each \;antibiotic \; (CFU/mL)}{Viable \; cells \; on \; MHA\;without \;antibiotic \;(CFU/mL)}$$

The PAP assay was performed in triplicate. Mean values of the frequency of resistant cell were plotted with the standard errors of the means represented by error bars. The heteroresistant subpopulation was defined as the frequency of resistant cells ≥ 10^–7^ at eightfold above the antibiotic concentration of the main population. The resistant phenotype without subpopulation was defined as the frequency of resistant cells < 10^–7^ at eightfold above the antibiotic concentration of the main population.

The stability of the heteroresistance phenotype was evaluated as previously described with a slight modification^49^. Briefly, A single colony grown on agar supplemented with eightfold above the antibiotic concentration of the main population (the heteroresistance) was inoculated in CAMHB and incubated 37 °C for 24 h with shaking at 120 rpm. A subculture (1:1000) was inoculated and incubated as a previous step twice. After 3 days, the PAP was determined at the eightfold antibiotic concentration of the main population of each isolate. The results were interpreted as the stable heteroresistance (the frequency of resistant at eightfold concentration of the main population < 10^–7^) and the unstable heteroresistance (the frequency of resistant at eightfold concentration of the main population ≥ 10^–7^).

### Statistical analysis

Statistical analysis was performed using GraphPad Prism version 5.0. The OMP expressions and mRNA expression of *adeB*, *adeE*, and *adeY* were performed in triplicated. Mean values of the relative expression were plotted with error bars representing the standard error of the mean (n = 3). The expressions of each couple of the species were compared and calculated using the unpaired two-tailed t-test (*, *p*-value ˂0.05; **, *p*-value < 0.01; ***, *p*-value < 0.001 and ns, non-significant). The mRNA expression of cell wall recycling genes and *abaF* after treatment with fosfomycin were performed in triplicated. Mean values of the relative expression were plotted with error bars representing the standard error of the mean (n = 3). The expressions after fosfomycin treatment were compared to these of no treatment. The expressions after fosfomycin treatment for 4 h were compared to these of the treatment for 12 h. The *p*-values were calculated using the one-way ANOVA, Dunnett’s multiple comparison test (*, *p*-value ˂0.05; **, *p*-value < 0.01; ***, *p*-value < 0.001 and ns, non-significant).

## Supplementary Information

Supplementary Information.

## Data Availability

All data generated or analyzed during this study are included in this published article and its supplementary information files.
