# Supplementary material for: Synergism of imipenem with fosfomycin associated with the active cell wall recycling and heteroresistance in Acinetobacter calcoaceticus-baumannii complex
Source: Sci Rep. 2022 Jan 7;12:230. doi: 10.1038/s41598-021-04303-7 (PMC8741973; doi:10.1038/s41598-021-04303-7)
Supplement: Supplementary file 1 — Supplementary Information. [file 41598_2021_4303_MOESM1_ESM.docx]

**Synergism of imipenem with fosfomycin associated with the active cell wall recycling and heteroresistance in *Acinetobacter calcoaceticus*-*baumannii* complex.**

**
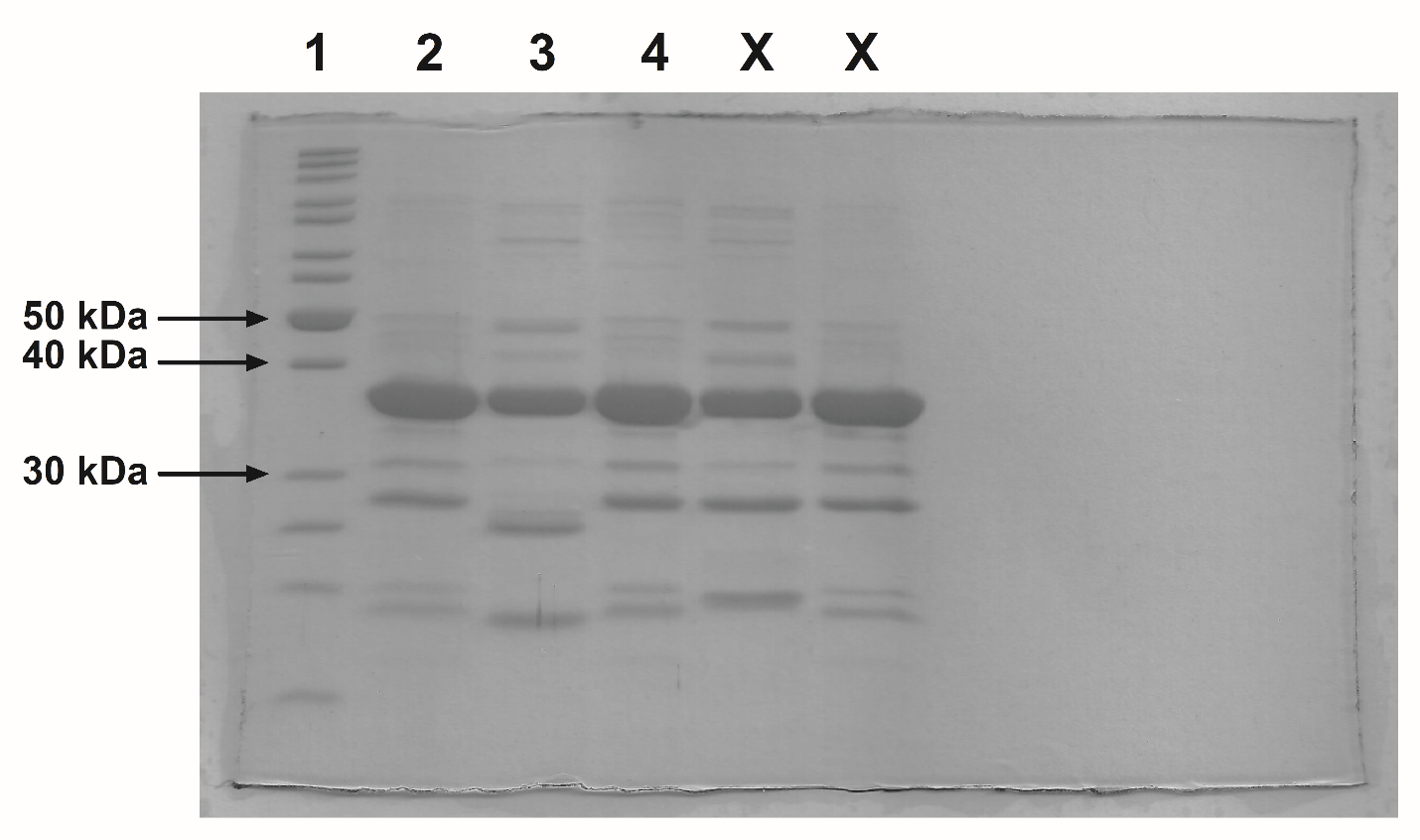
**

**Supplementary Figure S1A: Raw image of Figure 1A.** Outer membrane proteins (OMPs) of *A. baumannii* were analyzed by SDS-PAGE. The gels were dried on cellophane sheets. The OMP profiles were captured by an image scanner. 1: protein ladder, 2: *A. baumannii* ATCC 19606, 3: *A. baumannii* AB250, 4: *A. baumannii* A10, X: not included in Figure 1A.

**
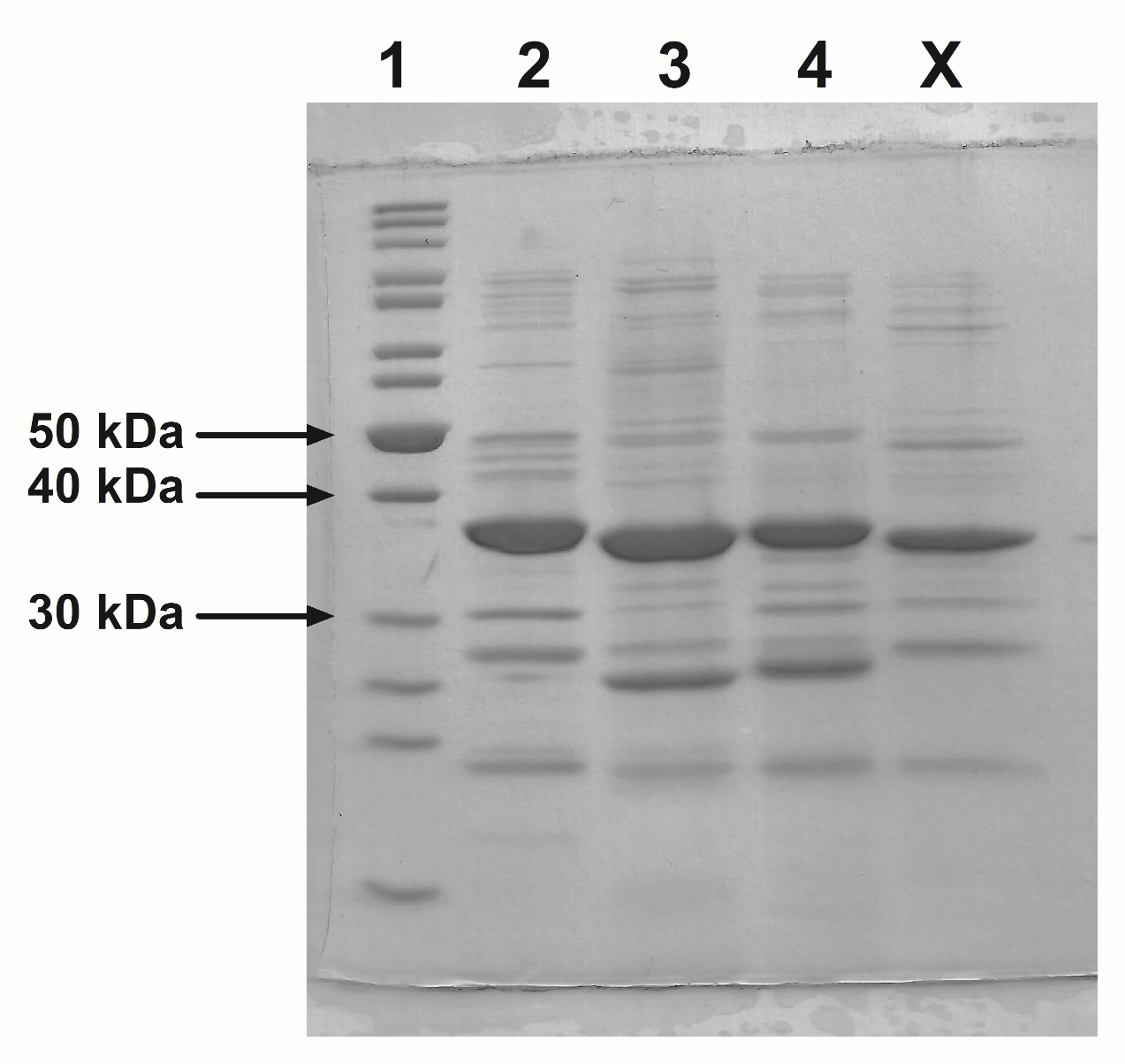
**

**Supplementary Figure S1B: Raw image of Figure 1B.** Outer membrane proteins (OMPs) of *A. baumannii* and *A. pittii* were analyzed by SDS-PAGE. The gels were dried on cellophane sheets. The OMP profiles were captured by an image scanner. 1: protein ladder, 2: *A. baumannii* ATCC 19606, 3: *A. pittii* AP1, 4: *A. pittii* AP23, X: not included in Figure 1B.

**
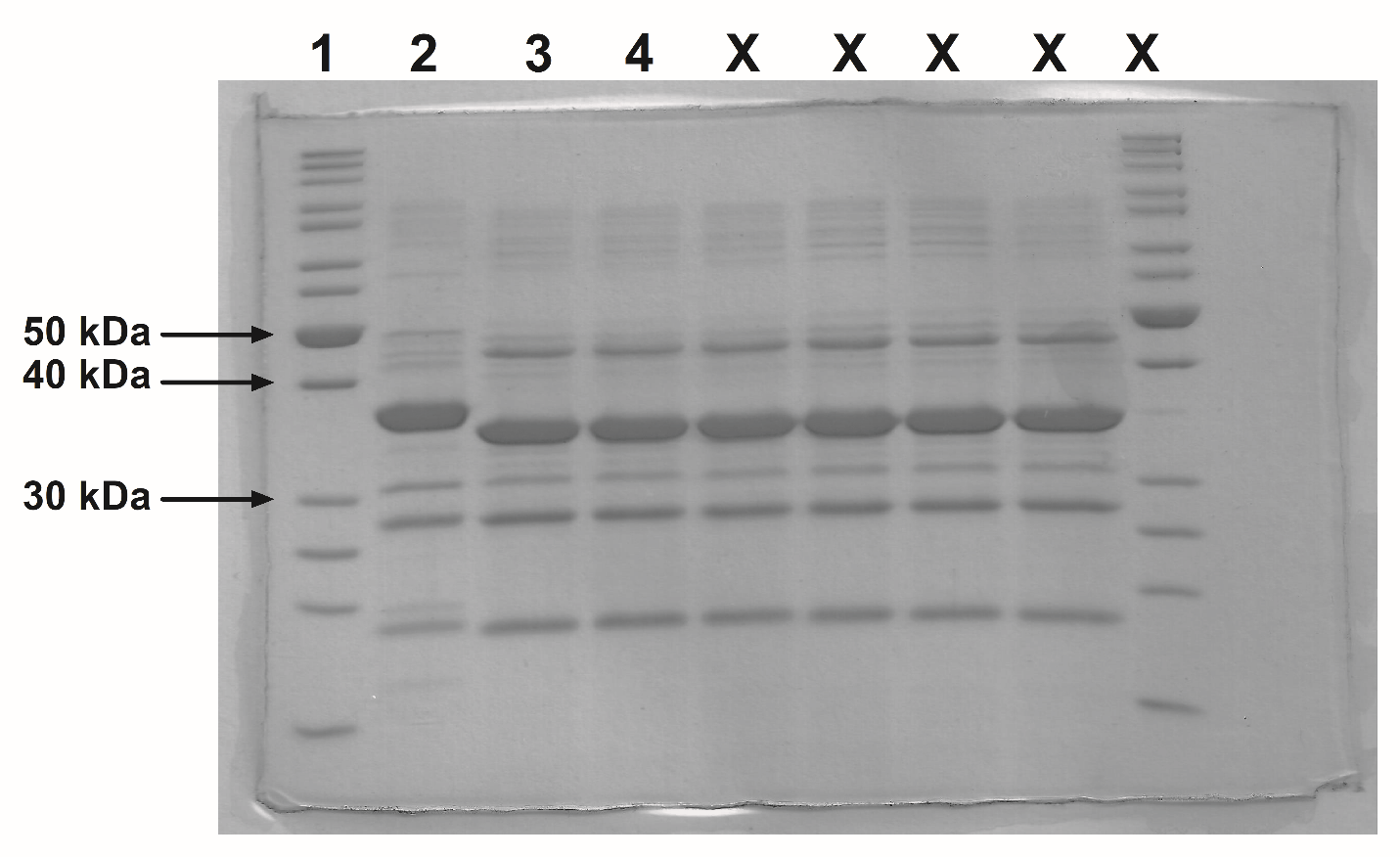
**

**Supplementary Figure S1C: Raw image of Figure 1C.** Outer membrane proteins (OMPs) of *A. baumannii* and *A. nosocomialis* were analyzed by SDS-PAGE. The gels were dried on cellophane sheets. The OMP profiles were captured by an image scanner. 1: protein ladder, 2: *A. baumannii* ATCC 19606, 3: *A. nosocomialis* AN4, 4: *A. nosocomialis* AN12, X: not included in Figure 1C.

**
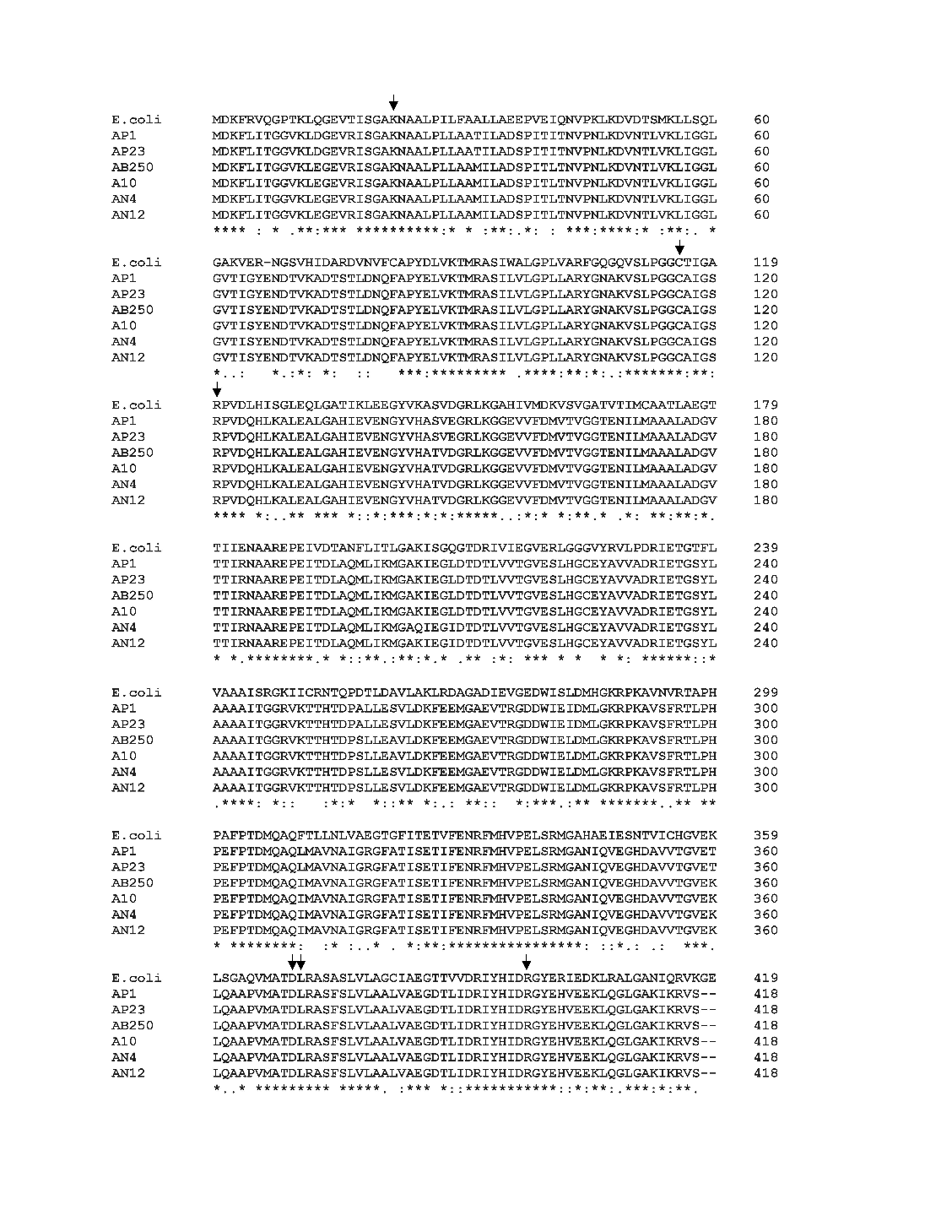
**

**Supplementary Figure S2:** Alignment of MurA amino acid sequences of fosfomycin-susceptible *E. coli* (Sequence ID: EFX33752.1) and six ACB isolates. Arrows represented the positions associated with fosfomycin resistance in *E. coli*.

**
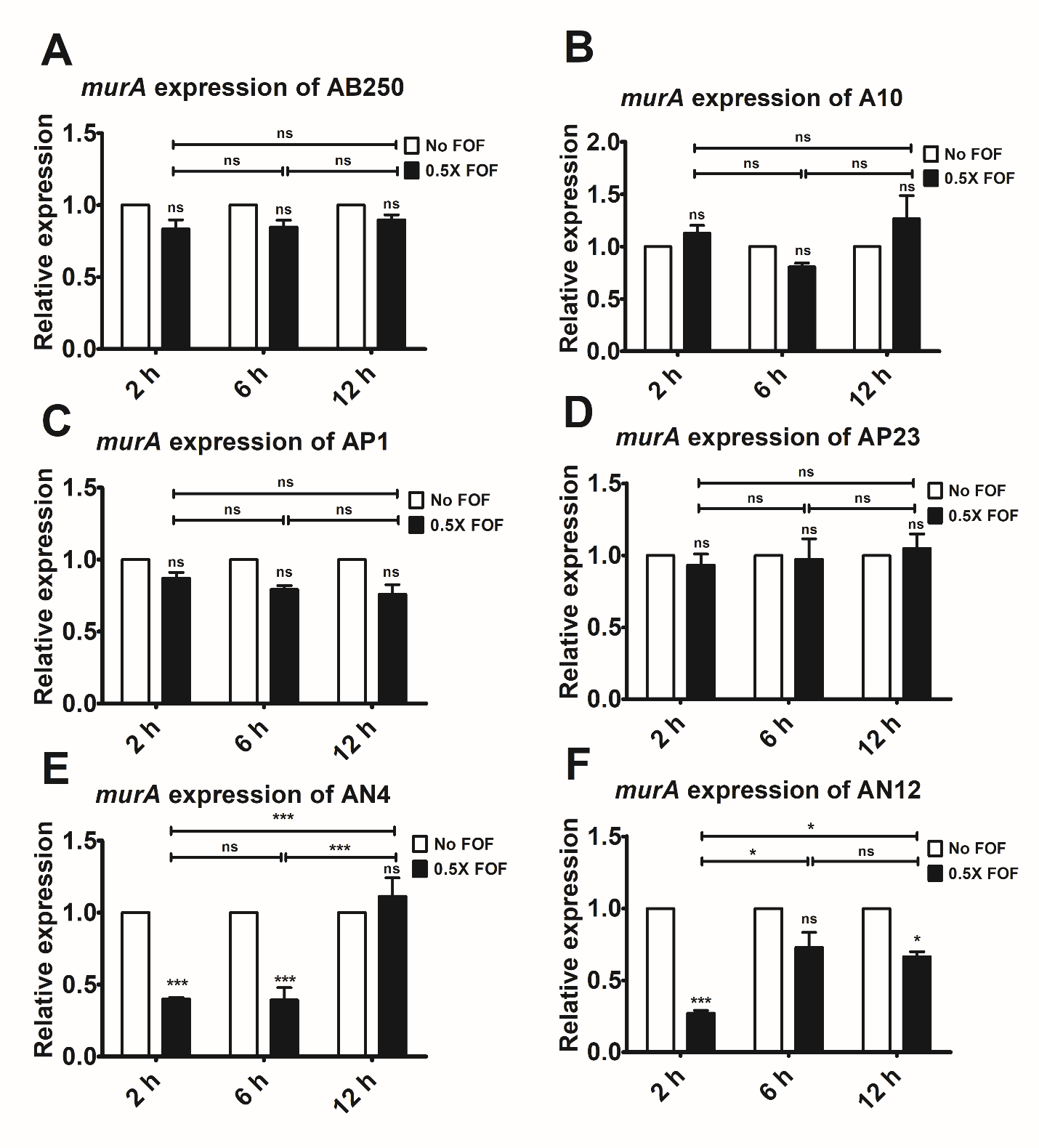
**

**Supplementary Figure S3:** Relative mRNA expression of *murA* in six ACB isolates. RT-PCR assay of *murA* expression after 2, 6, and 12 h of exposure to fosfomycin was determined in *A. baumannii* AB250 (A) and A10 (B), *A. pittii* AP1 (C) and AP23 (D), and *A. nosocomialis* AN4 (E) and AN12 (F). The relative mRNA expressions at each condition (in the presence of 0.5×MIC of fosfomycin) were normalized to 16S rRNA expression and compared to the mRNA expression level of each isolate in the absence of fosfomycin. All experiments were performed in triplicate. Mean values of the relative mRNA expression were plotted with error bars representing the standard error of the mean (n=3). The *p*-values were calculated using one-way ANOVA, Dunnett’s multiple comparison test (*, *p*-value ˂0.05; **, *p*-value <0.01; ***, *p*-value <0.001 and ns, non-significant).

**
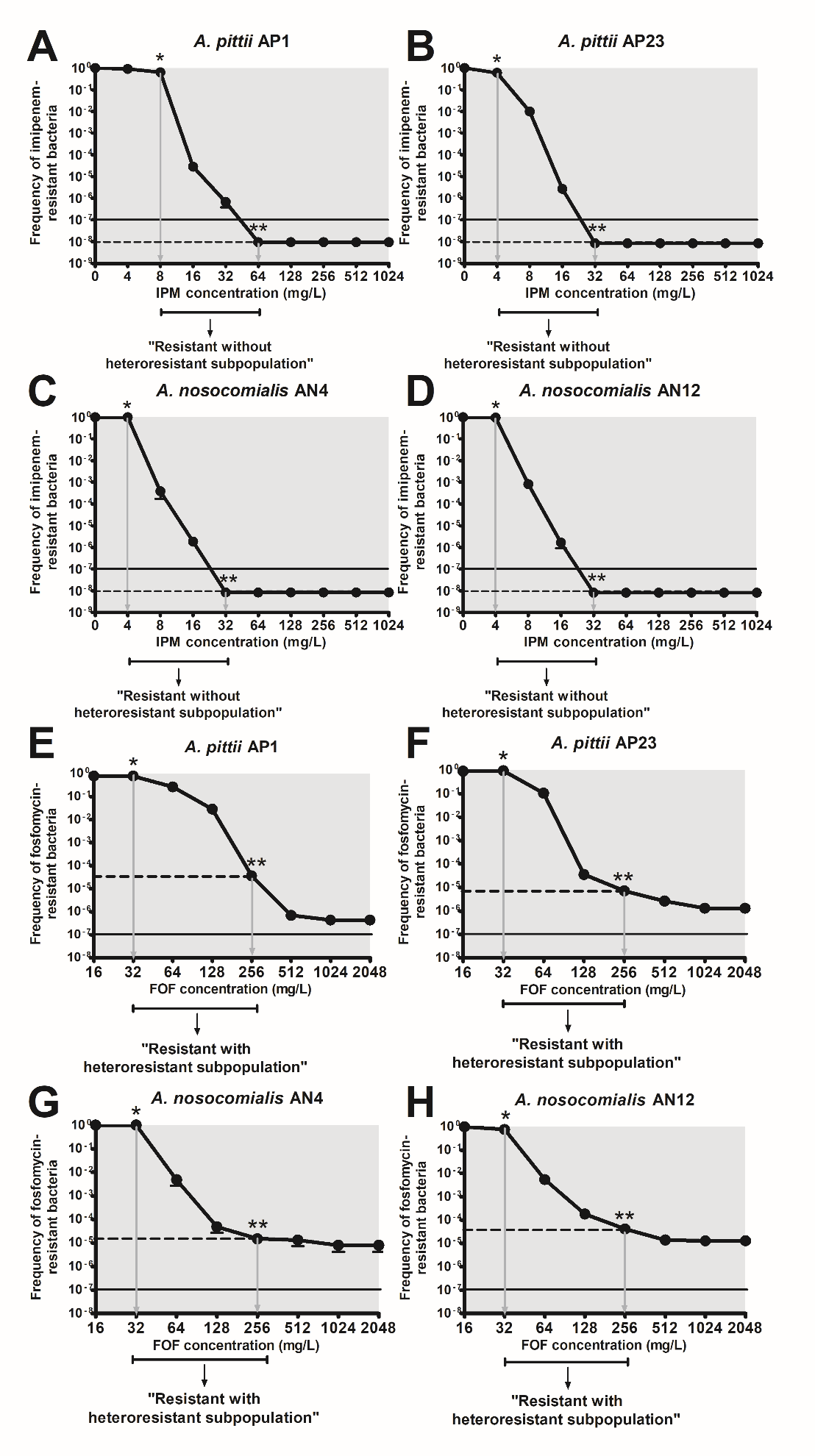
**

**Supplementary Figure S4:** PAP of imipenem and fosfomycin in *A. pittii* AP1 (A, E) and AP23 (B, F) and A. nosocomialis AN4 (C, G) and AN12 (D, H). The frequency of antibiotic-resistant bacteria was the relative of viable cells at each antibiotic concentration normalized to those in the absence of antibiotic. Mean values of the frequency of antibiotic-resistant bacteria were plotted with error bars representing the standard error of the mean (n=3). All experiments were performed in triplicate. The “resistant with heteroresistant subpopulation” was defined as that the frequency of antibiotic-resistant bacteria at 8-fold above the resistance level of the main population (dash lines) was higher than 10^-7^. *: antibiotic concentration (the resistance level) of the main population, **: 8-fold above antibiotic concentration (the resistance level) of the main population.

| **Antibiotic** | **Interpretation and MIC breakpoints (mg/L)** | | |
| --- | --- | --- | --- |
|  | **Susceptible (S)** | **Intermediate (I)** | **Resistant (R)** |
| **Imipenem (IPM)** | **≤2** | **4** | **≥8** |
| **Meropenem (MEM)** | **≤2** | **4** | **≥8** |
| **Amikacin (AMK)** | **≤16** | **32** | **≥64** |
| **Colistin (CT)** | **-** | **≤2** | **≥4** |
| **Fosfomycin (FOF)*** | **≤64** | **128** | **≥256** |

**Supplementary Table S1: The minimum inhibitory concentration (MICs) breakpoints and interpretation of *Acinetobacter* species.** *: interpretation for Enterobacterales (no interpretation for *Acinetobacter* species)

**Reference:** Clinical and Laboratory Standards Institute. Performance standards for antimicrobial susceptibility testing 30th informational supplement. Approved standard M100-S30. Clinical and Laboratory Standards Institute, Wayne (2020).

| **Isolate** | **IPM + AMK** | | | | | **IPM + CT** | | | | | **IPM + FOF** | | | | |
| --- | --- | --- | --- | --- | --- | --- | --- | --- | --- | --- | --- | --- | --- | --- | --- |
|  | **IPM** | **IPM com** | **AMK** | **AMK com** | **FICI** | **IPM** | **IPM com** | **CT** | **CT com** | **FICI** | **IPM** | **IPM com** | **FOF** | **FOF com** | **FICI** |
| ***A. baumannii* AB250** | 16 | 8 | 4 | 1 | **0.75** | 16 | 16 | 1 | 1 | **2.00** | 16 | 4 | 128 | 32 | **0.5** |
| ***A. baumannii***  **A10** | 128 | 64 | 2 | 0.5 | **0.75** | 128 | 64 | 2 | 0.5 | **0.75** | 128 | 32 | 256 | 64 | **0.5** |
| ***A. pittii***  **AP1** | 32 | 8 | 0.5 | 0.125 | **0.5** | 32 | 16 | 2 | 1 | **1.00** | 32 | 8 | 256 | 32 | **0.38** |
| ***A. pittii***  **AP23** | 16 | 8 | 2 | 0.5 | **0.75** | 16 | 8 | 1 | 0.5 | **1.00** | 16 | 4 | 128 | 32 | **0.5** |
| ***A. nosocomialis* AN4** | 16 | 8 | 2 | 0.25 | **0.63** | 16 | 16 | 2 | 2 | **2.00** | 16 | 4 | 256 | 64 | **0.5** |
| ***A. nosocomialis* AN12** | 32 | 8 | 2 | 0.25 | **0.38** | 32 | 16 | 2 | 1 | **1.00** | 32 | 8 | 128 | 32 | **0.5** |

**Supplementary Table S2: The minimum inhibitory concentration (MICs) of antibiotics used for calculation of the fractional inhibitory concentration index (FICI).** The MICs of imipenem (IPM), amikacin (AMK), colistin (CT), fosfomycin (FOF), and their MICs in the combination (com) with each antibiotic were obtained by checkerboard assay.

| **Isolate** | **MEM + AMK** | | | | | **MEM + CT** | | | | | **MEM + FOF** | | | | |
| --- | --- | --- | --- | --- | --- | --- | --- | --- | --- | --- | --- | --- | --- | --- | --- |
|  | **MEM** | **MEM com** | **AMK** | **AMK com** | **FICI** | **MEM** | **MEM com** | **CT** | **CT com** | **FICI** | **MEM** | **MEM com** | **FOF** | **FOF com** | **FICI** |
| ***A. baumannii* AB250** | 16 | 8 | 4 | 2 | **1.00** | 16 | 8 | 1 | 0.125 | **0.63** | 16 | 8 | 128 | 64 | **1.00** |
| ***A. baumannii***  **A10** | 256 | 128 | 2 | 1 | **1.00** | 256 | 128 | 2 | 1 | **1.00** | 256 | 128 | 256 | 64 | **0.75** |
| ***A. pittii***  **AP1** | 32 | 16 | 0.5 | 0.25 | **1.00** | 32 | 16 | 2 | 0.125 | **0.56** | 32 | 8 | 256 | 64 | **0.5** |
| ***A. pittii***  **AP23** | 32 | 16 | 2 | 0.25 | **0.63** | 32 | 16 | 1 | 0.5 | **1.00** | 32 | 16 | 128 | 64 | **1.00** |
| ***A. nosocomialis* AN4** | 32 | 16 | 2 | 0.5 | **0.75** | 32 | 16 | 2 | 0.5 | **0.75** | 32 | 8 | 256 | 64 | **0.5** |
| ***A. nosocomialis* AN12** | 64 | 32 | 2 | 0.5 | **0.75** | 64 | 64 | 2 | 2 | **2.00** | 64 | 16 | 128 | 32 | **0.5** |

**Supplementary Table S2 (continue): The minimum inhibitory concentration (MICs) of antibiotics used for calculation of the fractional inhibitory concentration index (FICI).** The MICs of meropenem (MEM), amikacin (AMK), colistin (CT), fosfomycin (FOF), and their MICs in the combination (com) with each antibiotic were obtained by checkerboard assay.

| **Isolate** | **Imipenem** | | | **Fosfomycin** | | |
| --- | --- | --- | --- | --- | --- | --- |
|  | **8-fold antibiotic of the main population (mg/L)** | **Frequency of the main population** | **Frequency of the heteroresistance**  **(Interpretation)** | **8-fold antibiotic of the main population**  **(mg/L)** | **Frequency of the main population** | **Frequency of the heteroresistance**  **(Interpretation)** |
| ***A. baumannii* AB250** | 32 | 8.57 × 10^-7^ | **1.67 × 10^-8^**  **(Unstable)** | 512 | 8.38 × 10^-7^ | **1.53 × 10^-8^**  **(Unstable)** |
| ***A. baumannii* A10** | 512 | 8.69 × 10^-9^ | **ND** | 1024 | 5.67 × 10^-7^ | **1.98 × 10^-8^**  **(Unstable)** |
| ***A. pittii* AP1** | 64 | 9.09 ×10^-9^ | **ND** | 256 | 5.58 × 10^-5^ | **2.85 ×10^-8^**  **(Unstable)** |
| ***A. pittii* AP23** | 32 | 7.88 ×10^-9^ | **ND** | 256 | 8.33 × 10^-6^ | **1.81× 10^-8^**  **(Unstable)** |
| ***A. nosocomialis* AN4** | 32 | 1.25 ×10^-8^ | **ND** | 256 | 2.30 × 10^-5^ | **1.25 × 10^-8^**  **(Unstable)** |
| ***A. nosocomialis* AN12** | 32 | 1.24 ×10^-8^ | **ND** | 256 | 6.10 × 10^-5^ | **1.25 ×10^-8^**  **(Unstable)** |

**Supplementary Table S3: The stability of the heteroresistance subpopulations to imipenem and fosfomycin.** The stability of the heteroresistance at 8-fold antibiotic concentration of the main population was evaluated by the PAP assay. ND: not determined.

| **Gene** | **Primer sequences** | **PCR product size (bp)** | **Application** | **Reference** |
| --- | --- | --- | --- | --- |
| *adeB* | F- TTAACGATAGCGTTGTAACC | 541 | PCR/ RT-PCR | Hou et al.^1^ |
|  | R- TGAGCAGACAATGGAATAGT |  |  |  |
| *adeE* | F- GTAGTAGTTCGGCAGGACAA | 376 | PCR/ RT-PCR | Chu et al.^2^ |
|  | R- GCGGTTCTAACATCTGATGG |  |  |  |
| *adeY* | F- CAATCTGCAACTGCGCTT | 587 | PCR/ RT-PCR | Chu et al.^2^ |
|  | R- TCAACAGCTTCTGCGGTA |  |  |  |
| *abaF* | F- ATCGGGATCCAGCAAAATTTGCACACTGTC | 732 | PCR/ RT-PCR | Sharma et al.^3^ |
|  | R- ATCGGGATCCTTGCAAAGAACCTATTAATCTAAAT |  |  |  |
| *murA* | F- TACGGAAATGCCAAGGTTTC | 232 | PCR/ RT-PCR | This study |
|  | R- TGGTTACACCATCTGCCAAA |  |  |  |
| *ampG* | F- ACAGGCGCAACTCAAGAT | 459 | PCR/ RT-PCR | Li et al.^4^ |
|  | R- CCCAATAAAGCAGCAACA |  |  |  |
| *murU* | F- ACGCTTTTGTACACCCAACC | 191 | PCR/ RT-PCR | This study |
|  | R- GTGCCCCATTTCCTCAACTA |  |  |  |
| 16S rRNA | F- GGAGGAAGGTGGGGATGACG | 241 | RT-PCR | Hou et al.^1^ |
|  | R- ATGGTGTGACGGGCGGTGTG |  |  |  |
| Entire *murA* | F- GAGGCGGAAGTAGTGGTGAG | 1764 | Sequencing | This study |
|  | R- GAGCGCCATGTTCCATAAGT |  |  |  |
| *nagZ* | F- TTTGATTGCTGTCGACCAAG | 240 | PCR/RT-PCR | This study |
|  | R- AAGCCACGGTCACCAATTAC |  |  |  |
| *anmK* | F- ATTCGAACCCGAGTTACGTG | 216 | PCR/RT-PCR | This study |
|  | R- AGCGTAAAACCATGCTCTGG |  |  |  |
| *amgK* | F- CTTTGGTGATGTGCTGCTGT | 210 | PCR | This study |
|  | R- TTTTGCTCAGCTGTTGGATG |  |  |  |

**Supplementary Table S4: Oligonucleotide sequences of primers used in this study.**

**References**

1. Hou, P.F., Chen, X.Y., Yan, G.F., Wang, Y.P. & Ying, C.M. Study of the correlation of imipenem resistance with efflux pump adeABC, AdeIJK, adeDE, and AbeM in clinical isolates of *Acinetobacter baumannii*. *Chemotherapy*. **58**, 152-158, doi:10.1159/000335599 (2012).
2. Chu, Y.W., Chau, S.L. & Houang, E.T. Presence of active efflux systems AdeABC, AdeDE, and AdeXYZ in different *Acinetobacter* genomic DNA groups. *J Med Microbiol*. **55**, 477-478, doi:10.1099/jmm.0.46433-0 (2006).
3. Sharma, A., Sharma, R., Bhattacharyya, T., Bhando, T., Pathania, R. Fosfomycin resistance in *Acinetobacter baumannii* is mediated by efflux through a major facilitator superfamily (MFS) transporter-AbaF. *J Antimicrob Chemother*. **72**, 68-74, doi:10.1093/jac/dkw382 (2017).
4. Li, P. et al. Structure-function analysis of the transmembrane protein AmpG from *Pseudomonas* *aeruginosa*. *PLoS One*. **12**, e0168060, doi:10.1371/journal.pone.0168060 (2016).
